# Comparing Internet-Based Cognitive Behavioral Therapy With Standard Care for Women With Fear of Birth: Randomized Controlled Trial

**DOI:** 10.2196/10420

**Published:** 2018-08-10

**Authors:** Elisabet Rondung, Elin Ternström, Ingegerd Hildingsson, Helen M Haines, Örjan Sundin, Johanna Ekdahl, Annika Karlström, Birgitta Larsson, Birgitta Segeblad, Rebecca Baylis, Christine Rubertsson

**Affiliations:** ^1^ Department of Psycholgy Mid Sweden University Östersund Sweden; ^2^ Department of Women’s and Children’s Health Uppsala University Uppsala Sweden; ^3^ Department of Nursing Mid Sweden University Sundsvall Sweden; ^4^ Department of Rural Health The University of Melbourne Victoria Australia; ^5^ Department of Obstetrics and Gynecology Uppsala University Hospital Uppsala Sweden; ^6^ Department of Health Science Faculty of Medicine Lund university Lund Sweden

**Keywords:** fear of birth, anxiety, pregnancy, cognitive behavioral therapy, internet-based

## Abstract

**Background:**

Although many pregnant women report fear related to the approaching birth, no consensus exists on how fear of birth should be handled in clinical care.

**Objective:**

This randomized controlled trial aimed to compare the efficacy of a guided internet-based self-help program based on cognitive behavioral therapy (guided ICBT) with standard care on the levels of fear of birth in a sample of pregnant women reporting fear of birth.

**Methods:**

This nonblinded, multicenter randomized controlled trial with a parallel design was conducted at three study centers (hospitals) in Sweden. Recruitment commenced at the ultrasound screening examination during gestational weeks 17-20. The therapist-guided ICBT intervention was inspired by the Unified protocol for transdiagnostic treatment of emotional disorders and consisted of 8 treatment modules and 1 module for postpartum follow-up. The aim was to help participants observe and understand their fear of birth and find new ways of coping with difficult thoughts and emotions. Standard care was offered in the three different study regions. The primary outcome was self-assessed levels of fear of birth, measured using the Fear of Birth Scale.

**Results:**

We included 258 pregnant women reporting clinically significant levels of fear of birth (guided ICBT group, 127; standard care group, 131). Of the 127 women randomized to the guided ICBT group, 103 (81%) commenced treatment, 60 (47%) moved on to the second module, and only 13 (10%) finished ≥4 modules. The levels of fear of birth did not differ between the intervention groups postintervention. At 1-year postpartum follow-up, participants in the guided ICBT group exhibited significantly lower levels of fear of birth (*U*=3674.00, *z*=−1.97, *P*=.049, Cohen *d*=0.28, 95% CI –0.01 to 0.57). Using the linear mixed models analysis, an overall decrease in the levels of fear of birth over time was found (*P*≤ .001), along with a significant interaction between time and intervention, showing a larger reduction in fear of birth in the guided ICBT group over time (*F*_1,192.538_=4.96, *P*=.03).

**Conclusions:**

Fear of birth decreased over time in both intervention groups; while the decrease was slightly larger in the guided ICBT group, the main effect of time alone, regardless of treatment allocation, was most evident. Poor treatment adherence to guided ICBT implies low feasibility and acceptance of this treatment.

**Trial Registration:**

ClinicalTrials.gov NCT02306434; https://clinicaltrials.gov/ct2/show/NCT02306434 (Archived by WebCite at http://www.webcitation.org/70sj83qat)

## Introduction

### Background

Fear of birth (FOB) has been recognized as an important component in psychosocial antenatal care. More than just affecting the emotional well-being of pregnant women, FOB has repeatedly been associated with measures of both anxiety and depression [1-3], as with obstetric complications [4], negative birth experiences [4,5], and requests for cesarean births [6,7].

Depending on the population studied and the measurement tool used for identification, the prevalence of FOB varies. However, a worldwide FOB prevalence of 14% has recently been found [8]. Commonly, primiparous women report slightly higher fear levels than multiparous women [9]. It remains unclear how levels of FOB change throughout pregnancy. Huizink [10] found that the mean levels of FOB decreased from early to midpregnancy and were elevated again in late pregnancy. On the individual level, however, different patterns, with fear levels increasing or decreasing during pregnancy, have been shown [11]. The distress experienced because of FOB can persist beyond giving birth. Women with FOB during pregnancy are at risk of still feeling that fear as long as 1 year postpartum or in a subsequent pregnancy [12,13].

Although widely acknowledged in clinical care, the concept of FOB remains poorly defined [14,15], and several terms such as fear of childbirth [16,17], tokophobia [18], or pregnancy anxiety [19] are being used. In essence, FOB refers to experiences of fear, anxiety, or worry related to giving birth. Little is known about the psychological constructs explaining FOB [9]. A distinction is commonly made between fear that predates first pregnancy—primary FOB—and fear that appears following traumatic or distressing childbirth—secondary FOB [18]. Many other variations exist among women fearing birth with regard to fear acquisition, fear objects, symptom severity, and comorbidity [20-23]. Thus, it is important that interventions for FOB are broad or adequately flexible to meet this heterogeneity.

No consensus exists on how FOB should be handled in clinical care. A few different treatment protocols have been evaluated in randomized controlled trials (RCTs). When comparing psychoeducational group sessions with standard care (SC) for women with severe FOB, fewer cesarean births, more positive birth experiences, and less depressive symptoms postpartum were found in the intervention group, however, with small effect sizes for the psychological variables [24-26]. An Australian trial compared midwife telephone counseling on two occasions to standard antenatal care, showing reduced levels of FOB at postintervention (gestational week 36) in both groups [27]. After adjustment for the preintervention levels of FOB, the reduction in FOB was slightly higher for women in the intervention group. In Sweden, women with FOB during pregnancy are, after referral, offered counseling by specially trained midwives [28]. Larsson et al [5] reported that 1 year postpartum most women who received this counseling for FOB were satisfied with their care; however, their pregnancies and births were less favorable than nonfearful women in terms of their level of fear, degree of positive birth experiences, and rate of elective cesarean births. Although generally showing some positive effects of interventions targeting FOB, none of these studies has been convincing in reducing FOB. Given the apparent associations between FOB and measures of anxiety and depression, treatment protocols known to be efficacious in reducing fear, anxiety, and depressive symptoms are thus important to explore.

To date, cognitive behavioral therapy (CBT) remains the treatment of choice for most anxiety disorders [29-33] and one of the treatment alternatives recommended for depression [34]. These recommendations apply to women in the antenatal and postpartum period [35].

Recent advances in the field of CBT offer two highly interesting treatment alternatives with regard to FOB. First, transdiagnostic CBT treatment protocols have shown to be as efficacious as diagnosis-specific interventions for anxiety disorders, with robust effects even in the presence of comorbidity [36]. Considering the various associations between FOB and measures of anxiety and depression, the lack of knowledge regarding specific psychological mechanisms underpinning FOB and the apparent heterogeneity with regard to the symptom severity, comorbidity, and anxious focus, a transdiagnostic approach to CBT might be especially suitable in this context. Second, while the evidence is not yet conclusive [37], evaluations of interventions building on the principles and techniques of CBT but provided over the internet suggest equivalency with face-to-face CBT in terms of efficacy [38]. Guided internet-based self-help programs are well accepted by patients [39] and can be advantageous with regard to patients’ access to treatment, the amount of therapist time required, and their cost-effectiveness [40]. These advantages could be important when trying to implement a new treatment approach in psychosocial antenatal care. Additionally, a treatment that is flexible with regard to time and location might well suit the needs of expecting mothers and families.

Although internet-based self-help based on the principles of CBT could hold promise as a treatment alternative for women experiencing FOB, only one earlier study investigating the feasibility of such an approach has been published. In this nonrandomized study, Nieminen et al [41] tested an internet-based CBT self-help program for primiparous women with FOB. The authors reported a within-group decrease in FOB from preintervention to postintervention (Cohen *d*=0.95) and concluded that internet-based cognitive behavioral therapy (ICBT) has potential in the treatment of FOB for motivated primiparous women; however, they recommended confirmation by randomized studies.

### Objective

The primary aim of this RCT was to evaluate the efficacy of a guided internet-based self-help program based on CBT compared with SC on the levels of FOB in late pregnancy and 1 year postpartum in a Swedish sample of primiparous and multiparous women reporting clinically significant levels of FOB.

## Methods

### Design and Setting

This RCT was associated with the Uppsala University Psychosocial Care Program (U-CARE). This study, called the U-CARE Pregnancy trial, was a nonblinded, multicenter RCT with a parallel design, comparing guided ICBT with SC for pregnant women reporting FOB [42]. It was registered at ClinicalTrials.gov (NCT02306434) and approved by the Regional Ethical Review Board in Uppsala (No. 2013/209). We used a study-specific website called the U-CARE portal [43] for data collection and implementation of the guided ICBT intervention. Once the study was launched, the methods used for data collection and internet-based intervention were frozen and could not be changed. Recruitment and SC interventions were conducted at three study centers in Sweden—1 university hospital with an annual rate of 4000 births and 2 referral hospitals with an annual rate of 2800 and 1600 births, respectively.

### Sample Size Estimation

The sample size was determined on a reduction in the level of FOB, assessed in midpregnancy and 1 year after giving birth. The sample size of this study was based on a Swedish study, where 59% of women who had FOB during pregnancy reported no FOB 1 year postpartum [44]. With a 20% reduction of FOB, a two-sided test, a power of.80, and a significance level of 5%, the power calculation showed that approximately 200 participants needed to be enrolled in this study [42].

### Participants

Between February 2014 and February 2015, women undergoing ultrasound screening examination in gestational weeks 17-20 were screened for possible identification of FOB. The level of FOB was assessed by the Fear of Birth Scale (FOBS), where a cutoff of ≥60 was used to identify FOB [11,45]. The inclusion criteria were an ongoing pregnancy in gestational weeks 17-20, an ultrasound screening examination with no reported adverse findings, FOBS≥60, proficiency in Swedish language, and personal access to a mobile phone and computer with internet connection. Before enrollment, eligible women were given written and oral information about the study by the research midwives, and women willing to participate gave their written informed consent. Those who gave their consent received log-in details to the U-CARE portal and logged in and completed the internet-based preintervention questionnaire. After the completion of the questionnaire, participants were randomized by the U-CARE portal (1:1) to either the guided ICBT or the SC group.

**Figure 1:**
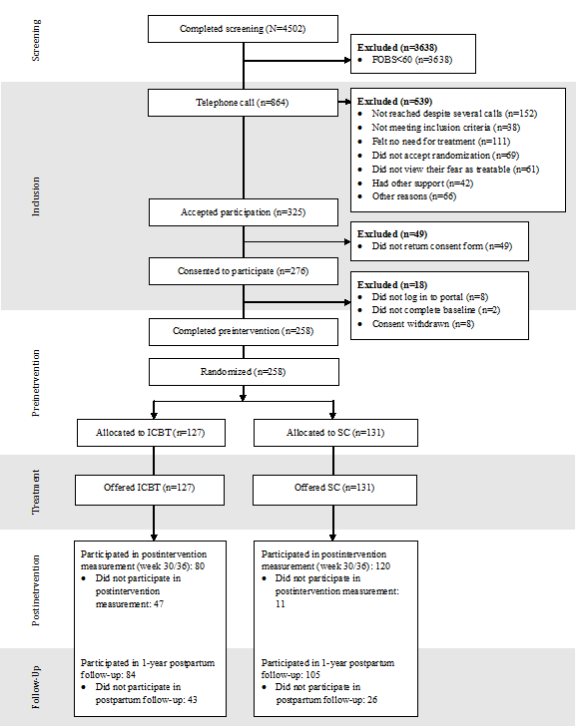
Flowchart of participants throughout the trial. ICBT: internet-based cognitive behavioral therapy SC: standard care.

Of 4502 women screened for FOB, 864 (19%) had a FOBS score of ≥60. Of 325 women who accepted participation in the RCT, 276 gave written informed consent. The main reasons for not willing to participate were that they felt no need for treatment (n=111), did not accept randomization (n=69), or felt that their fears (eg, fear of bleeding because of placenta previa, not finding an available hospital bed, or not reaching the hospital in time) could not be treated (n=61). In the end, 258 participants completed the preintervention questionnaire and were randomized as follows: 127 were allocated to the guided ICBT group and 131 to the SC group. Figure 1 shows the full Consolidated Standards of Reporting Trials flowchart.

### Guided Internet-Based Self-Help Program Based on Cognitive Behavioral Therapy

The aim of the guided ICBT intervention was to help participants observe and understand their FOB and find new ways of coping with difficult thoughts and emotions. With a study group that is likely to be highly heterogeneous (eg, with regard to parity and differences in fear acquisition, fear objects, symptom severity, and comorbidity), the treatment needed to be broad yet adequately flexible to be applicable to a wide range of different individual needs. Thus, the intervention was inspired by the unified protocol for transdiagnostic treatment of emotional disorders (UP), a broad face-to-face CBT protocol, designed for the applicability to all anxiety and unipolar mood disorders [46,47]. The study self-help material was built on the content of 7 of 8 modules in the UP, however, adapted to meet the needs of the current population (eg, with regard to the content and order of psychoeducative elements and by means of FOB-specific examples). Module 6 in the UP focuses specifically on the interoceptive exposure for induced symptoms. With a pregnant study sample, we decided to omit this module. To not risk getting into discussions of the accuracy of perceived threats, especially when not meeting participants face-to-face, we chose not to put too much emphasis on cognitive reappraisal (as presented in Module 4 in the UP). While still working on identifying automatic thoughts and giving some basic tools for reappraisal, we expanded the cognitive module by introducing exercises in cognitive defusion [48].

The self-help material was in Swedish and consisted of text material (81 downloadable PDF pages, including worksheets), audio files, photographs, and assignments related to each part of the program. The material was divided into 8 treatment modules and 1 module for the postpartum follow-up (see Textbox 1 for an overview). Participants were recommended to complete one self-help module per week. Each module included 1-3 homework assignments that were reported using the internet-based platform. On completion of the assignments, participants received personalized written feedback and were given access to the next self-help module.

**Overview of the guided internet-based self-help program based on cognitive behavioral therapy.**

Introduction and motivation enhancement

- Introduction to the program
- Understanding fear and anxiety
- Motivation and behavioral change
- Assignment: Setting individual treatment goals

Emotion

- The function of emotion
- Physiological, cognitive, and behavioral aspects of emotion
- Assignment: Self-monitoring of emotional reactions

Behavior

- Learned and emotion-driven behaviors
- Avoidance and negative reinforcement
- Assignment: Self-monitoring of emotion-driven behaviors and avoidance behaviors

Cognition

- Automatic appraisals and catastrophizing
- Viewing cognitions as merely cognitions: working with cognitive defusion
- Assignment: Identification of childbirth-related catastrophic cognitions
- Assignment: Cognitive defusion exercises

Mindfulness and acceptance

- Nonjudgmental present-moment awareness
- Acceptance in relation to pregnancy and childbirth
- Assignment: Guided present-focused awareness
- Assignment: Anchoring in the present
- Assignment: Identifying childbirth- or pregnancy-related areas in need of acceptance

Exposure, part I

- The purpose and value of exposure-based interventions
- Different forms of exposure: situational, imaginative, and interoceptive
- Assignment: Generating a personalized avoidance hierarchy for emotional exposure

Exposure, part II

- Planning and implementation of exposure-based interventions
- Assignment: Exposure to images related to childbirth
- Assignment: Exposure to avoided situations in accordance with personal hierarchy

Generalization and maintenance

- Progress and acquired skills: a summary of the program
- Being your own therapist: working with maintenance, relapse prevention, and further development
- Assignment: Evaluation of personal progress and acquired skills
- Assignment: Creating a plan for maintenance and future development

Postpartum follow-up

- Childbirth in retrospect: the unique experience of each childbirth
- Generalizing acquired skills to other areas of life
- Assignment: Reviewing the childbirth experience: cognitions, emotions, and strategies
- Assignment: Exposure to images related to childbirth
- Assignment: How can the acquired skills be generalized to other areas of life?

The guided ICBT program was delivered through a secure internet-based platform, the U-CARE portal, using double verification for log-in. When randomized to the guided ICBT group, participants were also randomized to one of the two licensed clinical psychologists, who guided them through the self-help program. A welcome message was sent to each participant in the portal, along with a short message service (SMS) text message to their mobile phone. Participants who did not log in or follow the treatment plan received reminders, both in the portal and through SMS text messages, at 10 days and 4 weeks after randomization or their last log-in. About half-time through the project, the psychologists started to call each participant randomized to the guided ICBT group to optimize adherence and motivation. In total, 37 participants talked with their psychologist on the phone, whereas 15 did not respond despite several calls. The psychologists were active in the U-CARE portal three times a week, giving feedback on homework assignments, sending reminders, and answering messages from participants in the U-CARE portal.

### Standard Care: Counseling by Midwives

All hospitals in Sweden provide SC for pregnant women with FOB [28]. Although guidelines exist [49], the content of SC and the time set aside for it differ between hospitals [28,50]. Women with FOB usually receive 2-4 counseling sessions either by antenatal midwives, counseling midwives and obstetricians, or a psychosocial unit consisting of midwives, obstetricians, and psychologists. The counseling aims at understanding the origin of fear, reducing the fear, preparing for childbirth, empowering women in their ability to give birth, and making the birth experience as positive as possible, regardless of the mode of birth [24]. Since SC is organized differently across the country, this also applies to SC at the study centers in this study. Depending on which study center a participant belonged to, SC started either in the next meeting with the antenatal midwife or after referral to a counseling midwife or a psychosocial unit [42].

### Primary Outcome Measure

In this study, the primary outcome measure was the levels of FOB, measured in late pregnancy using FOBS [11,45]. This 2-item 100-mm visual analog scale consists of the question “How do you feel right now about the approaching birth,” with the anchor words calm or worried and no fear or strong fear. FOBS has previously been used in child-bearing populations [45,51-53] and has been proposed as a valid instrument for measuring FOB in both research and clinical contexts [11,45]. The postpartum version of FOBS is worded as “How do you feel right now when thinking about giving birth again,” with the anchor words calm or worried and no fear or strong fear.

### Data Collection

Data were collected through self-assessment questionnaires at 4 time-points as follows: (1) at the ultrasound screening examination in gestational weeks 17-20; (2) through the U-CARE portal at preintervention in gestational weeks 20-25; (3) through the U-CARE portal at postintervention in gestational weeks 30 and 36; (4) through the U-CARE portal and offline questionnaires at follow-up, 1 year postpartum. Reminders were sent to each participant at 1, 6, 12, 30, and 38 days after the start of each time-point to maintain retention. Demographic and obstetric data were collected at preintervention. FOBS was included in all time-points.

### Statistical Analysis

Statistical analyses were informed by the Consolidated Standards of Reporting Trials checklist [54] and conducted in the SPSS Statistics for Windows, version 24 (IBM Corp., Armonk, NY). Data from gestational weeks 30 and 36 were combined using the last observation carried forward, and the parity variable was dummy coded (0=primiparous, 1=multiparous). Participants who did not respond at either postintervention or follow-up were defined as lost to follow-up. Between-group differences in preintervention characteristics were analyzed using the independent-sample *t* test and Mann-Whitney *U* test for continuous variables and the Pearson’s chi-square test for categorical variables. Little’s missing completely at random test [55] was used to conclude that data were missing completely at random.

In this intention-to-treat study, linear mixed model analyses were used to analyze changes in FOB over time and whether such changes were dependent on the treatment allocation, parity of participants, or the interaction of both. Building on a likelihood-based approach, the linear mixed models analysis uses all available data and produces unbiased parameter estimates under the assumption of data being missing at random, making it suitable for intention-to-treat analyses in longitudinal studies with data missing at random [56-58]. We used the maximum likelihood estimation to compare the first basic model with subsequent models of increasing complexity using the likelihood ratio statistic [59].

The linear mixed model analyses were conducted in a sequence of nested models. The basic model examined the fixed effect of time on the dependent variable FOB, with a fixed intercept. The time variable represented the pregnancy week in which women responded to the questionnaires, with the intercept (point of zero) being the estimated due date. In the second model, a random effect of time, with a random intercept was included, adopting an unstructured covariance structure. Two different variants of the third model were conducted, one including the fixed effects of treatment and treatment × time, the other including the fixed effects of parity and parity × time. The fourth model involved all parameters included in the third models, as well as the three-way interaction between time, treatment, and parity. We compared the improved fit of each model with the preceding one using the likelihood ratio statistic [59].

As the linear mixed model analysis provides individual estimates of the outcome variable for each model tested, the estimated means were calculated from the values predicted in the last model that was statistically superior to prior models. We analyzed the between-group differences in FOB at postintervention and 1-year follow-up using the Mann-Whitney *U* test. Next, we calculated between- and within-group effect sizes (Cohen *d*) and their 95% CIs on the basis of both observed and estimated data. The clinically significant reduction in FOB was calculated and defined by a cutoff 2 SDs below the preintervention mean of the group [60]. Differences in the rate of treatment responders between the intervention groups were compared using the Pearson’s chi-square test.

## Results

### Sample Characteristics

Table 1 presents the preintervention characteristics of study participants. The mean age of participants was 29.6 years (SD 4.88; range: 17-42 years), and 60% (154/258) of these were primiparous, whereas 40% (104/258) were multiparous. Regarding their preintervention characteristics, no difference was observed in the level of FOB between the parity groups. Primiparous women were younger (*P*<.001), and more often reported an eating disorder (*P*=.02), whereas multiparous women more often reported having had a previous miscarriage (*P*<.001) or abortion (*P*=.003; results not presented). Of the multiparous women, 36% (37/104) reported a previous negative birth experience, 22% (23/104) had experienced a previous emergency cesarean birth, and 25% (26/104) had experienced a birth aided by vacuum extraction. Of all participants, about 4% (10/258) were currently receiving CBT treatment, 11% (28/258) had participated in a CBT treatment prior to this pregnancy, and 7% (17/258) had received treatment for FOB prior to this pregnancy. The guided ICBT and SC groups did not differ with regard to any of the background characteristics or the level of FOB at screening or preintervention. Although all participants scored above the clinical cutoff for FOB at screening (FOBS ≥60), 52 (20%) scored below this cutoff at preintervention.

### Treatment Adherence

Table 2 shows the number of treatment modules opened by the participants in the guided ICBT group. Of all participants allocated to this intervention, 81% (103/127) commenced treatment. Among these, the mean time logged in the portal was 39.96 minutes (SD 49.88; range: 1-244 minutes) or 13.21 minutes per opened module (SD 10.03; range: 0.5-47). Primiparous and multiparous women did not differ with regard to any of the variables related to the treatment adherence. Feedback regarding the adherence to SC could not be retrieved from care providers. All participants randomized to the SC group did not report whether they received SC. Of 79 women responding to this question, 3 (4%) reported not having participating in any treatment. In accordance with the intention-to-treat principle, all participants were asked to complete postintervention and follow-up assessments, regardless of the treatment adherence.

### Missing Data Analysis

The Little’s missing completely at random test showed that data were missing completely at random in the primary outcome variable (χ^2^_8_=9.8, *P*=.28). Further analysis showed that participants defined as lost to follow-up (did not respond either at postintervention or follow-up) were no different from the other participants with regard to any preintervention characteristic or the level of FOB at screening or preintervention. However, participants lost to follow-up were more likely to belong to the guided ICBT group (χ^2^_1_=11.2, *P*<.001). Overall, 24 (18.9%) participants in the ICBT group and 7 (5.3%) in the SC group were lost to follow-up.

### Descriptive Statistics, Mean Differences, and Effect Sizes

**Figure 2:**
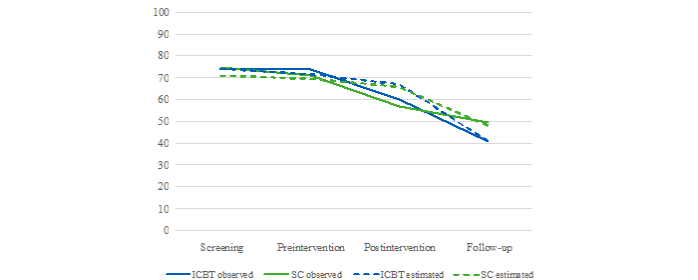
Observed and estimated Fear of Birth Scale mean scores from screening until 1 year postpartum. ICBT: internet-based cognitive behavioral therapy; SC: standard care.

Figure 2 plots and Tables 3 and 4 present the observed and estimated means and SDs of the primary outcome measure, along with the within-group effect sizes (Cohen *d*) and 95% CIs. The estimated means were calculated from the individual values of FOB predicted in Model 3a in the linear mixed model analysis. The levels of FOB did not differ between the intervention groups at postintervention. At 1-year postpartum follow-up, participants in the guided ICBT group exhibited significantly lower levels of FOB, both in the observed and estimated data (*U*=3674.00, *z*=–1.97, *P*=.049 and *U*=6985.00, *z*=–2.23, *P*=.027, respectively). Although the within-group effect sizes were generally found to be moderate or large, the between-group effect sizes were small or very small, Cohen *d*=0.14 favoring SC at postintervention and Cohen *d*=0.28 favoring the guided ICBT at follow-up in the observed data. The estimated between-group effect sizes were Cohen *d*=0.15 at postintervention, and Cohen *d*=0.29 at follow-up. At postintervention, 99 of 200 responding women had a FOBS score of ≥60 (guided ICBT group, 44/80; SC group, 55/120). At follow-up, the corresponding figures were 65 of 189 in the total sample (guided ICBT group, 29/84; SC group, 36/105).

### Responder Analysis

In line with recommendations by Jacobson and Truax [60], the cutoff for responding to treatment was set at 2 SDs below the preintervention mean of the group (FOBS≤38). At postintervention, a significantly higher proportion of participants in the SC group scored below this cutoff, 29 (22.1%) compared with 11 (8.7%) in the guided ICBT group (χ^2^_1_=8.9, *P*=.003). At follow-up, the groups did not differ significantly, with 44 (34.6%) participants in the guided ICBT group and 37 (28.2%) participants in the SC group reaching below this cutoff.

### Linear Mixed Model Analysis

The basic linear mixed model showed a significant effect of time on FOB. Overall, the FOBS score decreased from screening to follow-up (*F*_1,905_=220.08, *P*<.001).

**Table 1:** Characteristics of participants at preintervention.

Characteristics		Guided internet-based cognitive behavioral therapy (n=127), n (valid %)	Standard care (n=131), n (valid %)	All participants (n=258), n (valid %)	
**Age in years**					
	<25	21 (16.5)	16 (12.2)	37 (14.3)	
	25-35	90 (70.9)	96 (73.3)	186 (72.1)	
	>35	16 (12.6)	19 (14.5)	35 (13.6)	
**Civil status**					
	Living with partner	121 (95.3)	122 (93.1)	243 (94.2)	
	Not living with partner	6 (4.7)	9 (6.9)	15 (5.8)	
**Level of education**					
	Compulsory school or high school	55 (43.3)	65 (49.6)	120 (46.5)	
	University education	72 (56.7)	66 (50.4)	138 (53.5)	
**Country of birth**					
	Sweden	108 (85.0)	116 (88.5)	224 (86.8)	
	Other country	19 (15.0)	15 (11.5)	34 (13.2)	
**Computer illiterate**					
	Yes	5 (3.9)	4 (3.1)	9 (3.5)	
	No	122 (96.1)	127 (96.9)	249 (96.5)	
**Previous abortion**					
	Yes	31 (24.4)	30 (22.9)	61 (23.6)	
	No	96 (75.6)	101 (77.1)	197 (76.4)	
**Previous miscarriage**					
	Yes	30 (23.6)	30 (22.9)	60 (23.3)	
	No	97 (76.4)	101 (77.1)	198 (76.7)	
**Ongoing or history of depression**					
	Yes	41 (32.3)	50 (38.2)	91 (35.3)	
	No	86 (67.7)	81 (61.8)	167 (64.7)	
**Ongoing or history of anxiety**					
	Yes	35 (27.6)	40 (30.5)	75 (29.1)	
	No	92 (72.4)	91 (69.5)	183 (70.9)	
**Ongoing or history of an eating disorder **					
	Yes	14 (11.0)	16 (12.2)	30 (11.6)	
	No	113 (89.0)	115 (87.8)	228 (88.4)	
**Ongoing or history of bipolar disorder**					
	Yes	0 (0.0)	3 (2.3)	3 (1.2)	
	No	127 (100.0)	128 (97.7)	255 (98.8)	
**Ongoing or history of other psychiatric disorder **					
	Yes	7 (5.5)	15 (11.5)	22 (8.5)	
	No	120 (94.5)	116 (88.5)	236 (91.5)	
**Using medication for depression or anxiety at present**					
	Yes	7 (5.5)	13 (9.9)	20 (7.8)	
	No	120 (94.5)	118 (90.1)	238 (92.2)	

**Table 2:** Participants in the guided internet-based cognitive behavioral therapy (ICBT) group who opened each treatment module and the mean time spent per module.

Module	ICBT group (n=127)		Primiparas (n=77)		Multiparas (n=50)	
	Opened module	Minutes in module^a^	Opened module	Minutes in module^a^	Opened module	Minutes in module^a^
	n (%)	Mean	n (%)	Mean	n (%)	Mean
1	103 (81)	12.49	64 (83)	12.56	39 (78)	12.36
2	60 (47)	10.97	36 (47)	11.17	24 (48)	10.67
3	35 (28)	29.49	23 (30)	31.00	12 (24)	26.58
4	24 (19)	22.38	19 (25)	22.47	5 (10)	22.00
5	13 (10)	35.62	10 (13)	34.80	3 (6)	38.33
6	7 (6)	12.86	5 (6)	9.60	2 (4)	21.00
7	1 (1)	29.00	0 (0)	0.00	1 (2)	29.00
8	1 (1)	20.00	0 (0)	0.00	1 (2)	20.00
9^b^	1 (1)	1.00	0 (0)	0.00	1 (2)	1.00

^a^Mean time, measured in minutes, spent per module by participants who opened the module.

^b^Module for the postpartum follow-up.

**Table 3:** Observed means and SDs of Fear of Birth Scale scores at screening, preintervention, postintervention, and follow-up, including the within-group effect sizes.

Type of Intervention		Descriptive statistics		Effect size	
		n	Mean (SD)	Cohen *d*	95% CI
**Guided ICBT^a^**					
	Screening	127	74.76 (10.38)		
	Preintervention	127	74.06 (16.70)		
	Postintervention	80	60.56 (21.63)	0.58^b^	0.26-0.89
	Follow-up	84	41.17 (32.65)	1.23^c^	0.89-1.55
**Standard Care**					
	Screening	131	74.96 (11.36)		
	Preintervention	131	71.44 (17.99)		
	Postintervention	120	57.20 (24.83)	0.70^d^	0.44-0.96
	Follow-up	105	50.11 (30.48)	0.86^e^	0.58-1.14

^a^ICBT: internet-based cognitive behavioral therapy.

^b^Preintervention (n=80) mean 71.80 (SD 16.88).

^c^Preintervention (n=84) mean 72.92 (SD 16.49).

^d^Preintervention (n=120) mean 72.20 (SD 17.47).

^e^Preintervention (n=80) mean 71.58 (SD 17.69).

**Table 4:** Estimated means and SDs of the Fear of Birth Scale scores at screening, preintervention, postintervention, and follow-up, including the within-group effect sizes.

Type of Intervention		Descriptive statistics		Effect size	
		n	Mean (SD)	Cohen *d*	95% CI
**Guided ICBT^a^**					
	Screening	127	74.26 (5.73)		
	Preintervention	127	71.76 (6.61)		
	Postintervention	127	67.15 (8.62)	0.60	0.35-0.85
	Follow-up	127	41.03 (22.45)	1.86	1.56-2.14
**Standard Care**					
	Screening	131	70.94 (6.33)		
	Preintervention	131	69.29 (7.33)		
	Postintervention	131	65.73 (9.65)	0.42	0.17-0.66
	Follow-up	131	47.87 (24.10)	1.20	0.94-1.46

^a^ICBT: internet-based cognitive behavioral therapy.

The second model, examining whether the effect of time on FOB differed between individuals, showed significantly better fit with data than the first model (χ^2^_3_=214.4, *P*<.001). Significant variance remaining in the intercept and time variable indicated significant differences between participants with regard to the estimated level of FOB at the estimated due date and in the effect of time on FOB. Overall, this implies that both levels of FOB and how these levels changed over time differed significantly between participants.

In the third pair of models, we investigated whether these individual differences in the effect of time on FOB could be attributed to the treatment allocation or parity. In Model 3a, a significant interaction between treatment and time was found (*F*_1,192.538_=4.96, *P*=.03), showing that the reduction in FOB over time was significantly larger in the guided ICBT group (−0.46 units/week) than in the SC group (−0.31 units/week). However, the predicted level of FOB at the estimated due date did not differ significantly (guided ICBT group, 64.61; SC group, 64.15; *t*_1,240.996_=−0.24, *P*=.81). Hence, when comparing the intervention groups, no difference was observed in FOB in late pregnancy. When considering the entire study period, FOB decreased more in the guided ICBT group. In comparison with Model 2, Model 3a showed significantly better fit with data (χ^2^_2_=7.8, *P*=.02). However, Model 3b did not show a better fit with data than Model 2 (χ^2^_2_=0.5, *P*=.80), with no significant interaction between parity and time. Hence, changes in FOB over time were not significantly different between primiparous and multiparous women.

The main purpose of Model 4 was to examine the possibility of a three-way interaction between time, treatment, and parity. No such interaction effect was found, and Model 4 did not show a better fit with data compared with Model 3a (χ^2^_4_=0.5, *P*=.97), suggesting that the interaction between time and treatment did not differ depending on parity.

## Discussion

### Principal Findings

In this study, the level of FOB was found to decrease over time in both groups, generally with medium within-group effect sizes during pregnancy and large effect sizes from midpregnancy to 1 year postpartum. Similar decreases in FOB over time have been shown before, both among women with mixed levels of fear in early pregnancy [11] and among women receiving support for FOB [27]. This apparent effect of time alone points to the importance of including a proper control group when evaluating treatments for FOB. It is, thus, possible that the previously shown within-group effect of internet-based, therapist-supported, self-help for FOB [41] could, at least, in part, be attributable to a natural decrease in FOB over time. Unexpectedly, in 20% of participants, we observed a reduction of FOB below the inclusion criteria cutoff already before randomization and the introduction of any planned intervention. Although possibly an effect of the passage of time alone, this reduction might also be related to participants talking to a research midwife on the phone to be included in the study or simply because of statistical regression to the mean. As pregnancy itself is time-limited and the utmost feared situation will ultimately occur, the passage of time might have a unique meaning within this particular population.

When comparing the different interventions, participants allocated to the SC group were more likely to have responded to the treatment at postintervention measurement. However, mean differences were not significant at this time, and the between-group effect size was ignorable (Cohen *d*=0.14). In the linear mixed model analyses, a small, yet significant, interaction between time and treatment was found, indicating that over time FOB decreased slightly more among participants allocated to the guided ICBT group. This effect was most evident at 1-year postpartum follow-up, when participants in the guided ICBT group exhibited significantly lower levels of FOB, however, still with a small between-group effect size (Cohen *d*=0.28). This finding is not easily interpreted. First, given the low adherence to treatment in the guided ICBT group, these differences might not be attributable to the ICBT intervention. Perhaps, the differences rather relate to the interventions provided in the SC group. If so, our results might be in line with recently published results showing that women who receive SC for FOB still have higher levels of FOB in late pregnancy than women with FOB who do not receive SC [11]. Although highly valued by women receiving this form of support [5,61], the design of interventions provided in SC might be more focused on reducing FOB during the ongoing pregnancy (eg, by means of being able to convey what feels important during birth or planning for pain relief) than on treating fear in a long-term perspective.

Second, as the difference between the intervention groups does not appear until 1 year postpartum, multiple factors during childbirth and in the postpartum period might mediate this effect. Although findings are not coherent [62], previous research suggests that FOB during pregnancy might be positively correlated with the experience of pain during childbirth [63,64], longer birth duration [65-67], and a more negative rating of the overall birth experience [4,5]. Some studies have suggested a higher number of emergency cesarean births among women with FOB [68-70]. Furthermore, postpartum levels of FOB have been associated with previous mode of delivery, intervention at birth, or emergency cesarean births [4,13,20], as with more negative birth experiences [4,13]. Overall, when trying to understand FOB in the postpartum period, outcomes and experiences of giving birth are likely to contribute significantly, either as mediators, moderators, or as confounding variables. In this study, randomization and the resulting equivalence between the intervention groups prior to intervention will contribute to the minimization of the effect of extraneous variables.

In this trial, both primiparous and multiparous women were included. Although these groups are commonly separated and assumed to be in need of different interventions, there is nothing in our results that points specifically to that conclusion. We did not find any difference in FOB or the effect of the different interventions between primiparas and multiparas. Moreover, no difference was observed with regard to the treatment adherence or participant dropout. Hence, as far as we can see, none of these treatment alternatives seems to suit either parity group better.

### Limitations

This study has several methodological limitations—the most problematic of these related to the poor treatment adherence, participants being lost to follow-up, and wide inclusion criteria giving room for sample heterogeneity.

Concerning the SC group, we have no information on who actually received any counseling, how many appointments each participant had, who conducted the counseling or what it consisted of. We can only rely on the results of Larsson et al [28], showing that counseling exists nationwide but differs considerably in aspects such as available treatment options for women and educational background and time set aside for health care professionals providing counseling.

In the guided ICBT group, very low treatment adherence is obvious. Unfortunately, it is difficult to know all the reasons participants had for not engaging in their ICBT. Some participants reported reduced levels of fear, changes in their life circumstances, having received other forms of treatment, not having sufficient time, or problems related to the internet-based portal, whereas most did not respond to any attempt from the study team to get in contact. In this study, we did not measure the treatment acceptance or credibility. However, it seems likely to assume that the guided ICBT was not a well-accepted intervention in this sample. Quite a few potential participants declined participation because they did not accept randomization to either intervention, that is, they preferred SC beforehand. The 24 participants who did not commence the guided ICBT at all are likely to belong to that group as well. Fewer than half of the participants allocated to the guided ICBT group finished the first module and went on to the second module, and less than one-third advanced to Module 3. This poor adherence can be attributed to several possible reasons, potentially related to expectations and care preferences in the population, the process of inclusion and exclusion of participants, lack of preintervention assessment of individual needs, issues related to the U-CARE portal, instructions and reminders not being sufficient, treatment format, or self-help material not meeting the expectations of participants.

Besides low treatment adherence, there were quite large amounts of missing data, resulting in the need to combine two postintervention measures. Unfortunately, the amount of missing data was particularly evident in the guided ICBT group, presumably because of the low treatment adherence and participants having difficulties in differentiating between their treatment and data collection. However, with data being missing completely at random, we could use all available data and perform the intention-to-treat analysis using linear mixed models with the maximum likelihood estimation.

### Contribution

Although this study has some apparent limitations, it also has strengths. First, to the best of our knowledge, this is the first study using a randomized controlled design to evaluate the effects of CBT on FOB. The randomized controlled design and the equivalence between the intervention groups are important factors enhancing the internal validity of a study [71]. The inclusion of a control group is important to differentiate between the effect of the intervention and confounding variables. Although wait-list controls are commonly used in psychotherapy research, the limited time of pregnancy makes this control condition difficult to apply with regard to FOB. Although SC is difficult to control and thus may threaten the internal validity of the study, we still found this the most appropriate control condition available. Importantly, it gave us the possibility to differentiate the effect of treatment with what appears to be the effect of time alone.

Second, although primarily an efficacy study, the generous inclusion of participants and the naturalistic setting of this study resemble the prioritizations of effectiveness studies [71], resulting in a reduced level of control regarding potential confounding variables (eg, heterogeneity within the sample in terms of the symptom severity, comorbidity, and concurrent treatments). Despite these problems, the results still give a hint of how guided ICBT could work in a naturalistic setting. Since participants were included when visiting standard antenatal care, they have not actively asked for treatment for their FOB. Hence, the sample is not likely to be comparable with highly motivated samples of participants who have self-recruited to guided ICBT, as in the study by Nieminen et al [41] and many studies evaluating CBT delivered over the internet [72]. Instead, with regard to their age, civil status, and level of education, participants in this study were very similar to the general birthing population in Sweden [73]. As women with insufficient knowledge in the Swedish language were excluded from this study, the results cannot be generalized to this population.

### Conclusions

In this study, FOB decreased during pregnancy and until 1 year postpartum, both in the guided ICBT group and the SC group. The reduction in FOB was similar in the intervention groups during pregnancy, and the effect of time alone appeared as more important than the specific effect of any intervention. One year postpartum, a stronger reduction in FOB was found in the guided ICBT group—a finding that was not easily interpreted given the low adherence to the guided ICBT and the wide array of potential mediators, moderators, and confounders during childbirth and the postpartum period. Hence, the guided ICBT, as offered in this study, did not seem to be a feasible or well-accepted approach for treating FOB.

### Future Directions

The challenge in future research will be to find an intervention that is both well accepted by pregnant women and effective in reducing FOB. Considering the strong evidence for CBT in treating anxiety, cognitive and behavioral interventions should not be ruled out at this early point. However, to enhance the credibility among pregnant women and caregivers, we need to learn more about the experiences of women participating in different intervention programs and make adjustments in the treatment format and structure based on their views. Instead of comparing different treatment interventions, it might be more fruitful to integrate existing and well-accepted midwife counseling with CBT interventions, acknowledging the need for individual tailoring.

## Appendix

Multimedia Appendix 1 CONSORT-EHEALTH checklist (V 1.6.1).

## Abbreviations

CBT: cognitive behavioral therapy
FOB: fear of birth
FOBS: Fear of Birth Scale
ICBT: internet-based cognitive behavioral therapy
RCT: Randomized controlled trial
SC: standard care
U-CARE: Uppsala University Psychosocial Care Program
UP: unified protocol
