# Supplementary material for: Comparing Internet-Based Cognitive Behavioral Therapy With Standard Care for Women With Fear of Birth: Randomized Controlled Trial
Source: JMIR Ment Health. 2018 Aug 10;5(3):e10420. doi: 10.2196/10420 (PMC6109226; doi:10.2196/10420)
Supplement: Multimedia Appendix 1 [file mental_v5i3e10420_app1.pdf]

CONSORT-EHEALTH (V 1.6.1) - Submission/Publication Form

The CONSORT-EHEALTH checklist is intended for authors of randomized trials evaluating web-based and Internet-based applications/interventions, including mobile interventions, electronic games (incl multiplayer games), social media, certain telehealth applications, and other interactive and/or networked electronic applications. Some of the items (e.g. all subitems under item 5 - description of the intervention) may also be applicable for other study designs.

The goal of the CONSORT EHEALTH checklist and guideline is to be
a) a guide for reporting for authors of RCTs,
b) to form a basis for appraisal of an ehealth trial (in terms of validity)

CONSORT-EHEALTH items/subitems are MANDATORY reporting items for studies published in the Journal of Medical Internet Research and other journals / scientific societies endorsing the checklist.

Items numbered 1., 2., 3., 4a., 4b etc are original CONSORT or CONSORT-NPT (non-pharmacologic treatment) items.
Items with Roman numerals (i., ii, iii, iv etc.) are CONSORT-EHEALTH extensions/clarifications.

As the CONSORT-EHEALTH checklist is still considered in a formative stage, we would ask that you also RATE ON A SCALE OF 1-5 how important/useful you feel each item is FOR THE PURPOSE OF THE CHECKLIST and reporting guideline (optional).

Mandatory reporting items are marked with a red *.
In the textboxes, either copy & paste the relevant sections from your manuscript into this form - please include any quotes from your manuscript in QUOTATION MARKS,
or answer directly by providing additional information not in the manuscript, or elaborating on why the item was not relevant for this study.

YOUR ANSWERS WILL BE PUBLISHED AS A SUPPLEMENTARY FILE TO YOUR PUBLICATION IN JMIR AND ARE CONSIDERED PART OF YOUR PUBLICATION (IF ACCEPTED).
Please fill in these questions diligently. Information will not be copyedited, so please use proper spelling and grammar, use correct capitalization, and avoid abbreviations.

DO NOT FORGET TO SAVE AS PDF _AND_ CLICK THE SUBMIT BUTTON SO YOUR ANSWERS ARE IN OUR DATABASE !!!

Citation Suggestion (if you append the pdf as Appendix we suggest to cite this paper in the caption):
Eysenbach G, CONSORT-EHEALTH Group
CONSORT-EHEALTH: Improving and Standardizing Evaluation Reports of Web-based and Mobile Health Interventions
J Med Internet Res 2011;13(4):e126
URL: http://www.jmir.org/2011/4/e126/
doi: 10.2196/jmir.1923
PMID: 22209829

*Obligatorisk

Ditt svar är för stort. Testa att förkorta en del av dina svar.

Your name *

First Last

Elisabet Rondung

Primary Affiliation (short), City, Country *

University of Toronto, Toronto, Canada

Mid Sweden University, Östersund, Swed

Your e-mail address *

abc@gmail.com

elisabet.rondung@miun.se

Title of your manuscript *

Provide the (draft) title of your manuscript.

A randomized controlled trial comparing Internet-based cognitive behavior therapy with standard care for women with fear of birth

Name of your App/Software/Intervention *

If there is a short and a long/alternate name, write the short name first and add the long name in brackets.

U-CARE: Pregnancy

Evaluated Version (if any)

e.g. "V1", "Release 2017-03-01", "Version 2.0.27913"

Ditt svar

Language(s) *

What language is the intervention/app in? If multiple languages are available, separate by comma (e.g. "English, French")

Ditt svar är för stort. Testa att förkorta en del av dina svar.

URL of your Intervention Website or App *

e.g. a direct link to the mobile app on app in appstore (itunes, Google Play), or URL of the website. If the intervention is a DVD or hardware, you can also link to an Amazon page.

https://www.u-care.se/

URL of an image/screenshot (optional)

Ditt svar

Accessibility *

Can an enduser access the intervention presently?

- ○ access is free and open
- ◉ access only for special usergroups, not open
- ○ access is open to everyone, but requires payment/subscription/in-app purchases
- ○ app/intervention no longer accessible
- ○ Övrigt:

Primary Medical Indication/Disease/Condition *

e.g. "Stress", "Diabetes", or define the target group in brackets after the condition, e.g. "Autism (Parents of children with)", "Alzheimers (Informal Caregivers of)"

Fear of birth (Pregnant women with)

Primary Outcomes measured in trial *

comma-separated list of primary outcomes reported in the trial

Fear of Birth in late pregnancy

Secondary/other outcomes

Are there any other outcomes the intervention is expected to affect?

Fear of Birth one year after birth

Ditt svar är för stort. Testa att förkorta en del av dina svar.

Recommended "Dose" *

What do the instructions for users say on how often the app should be used?

- ○ Approximately Daily
- ○ Approximately Weekly
- ○ Approximately Monthly
- ○ Approximately Yearly
- ○ "as needed"
- ◉ Övrigt: One ICBT-module per week

Approx. Percentage of Users (starters) still using the app as recommended after 3 months *

- ○ unknown / not evaluated
- ○ 0-10%
- ○ 11-20%
- ○ 21-30%
- ○ 31-40%
- ○ 41-50%
- ○ 51-60%
- ○ 61-70%
- ○ 71%-80%
- ○ 81-90%
- ○ 91-100%
- ◉ Övrigt: Not applicable. Most participants are no longer pregnant after 3 mo

Ditt svar är för stort. Testa att förkorta en del av dina svar.

Overall, was the app/intervention effective? *

- ( ) yes: all primary outcomes were significantly better in intervention group vs control
- ( ) partly: SOME primary outcomes were significantly better in intervention group vs control
- ( ) no statistically significant difference between control and intervention
- ( ) potentially harmful: control was significantly better than intervention in one or more outcomes
- (x) inconclusive: more research is needed
- ( ) Övrigt:

Article Preparation Status/Stage *

At which stage in your article preparation are you currently (at the time you fill in this form)

- ( ) not submitted yet - in early draft status
- (x) not submitted yet - in late draft status, just before submission
- ( ) submitted to a journal but not reviewed yet
- ( ) submitted to a journal and after receiving initial reviewer comments
- ( ) submitted to a journal and accepted, but not published yet
- ( ) published
- ( ) Övrigt:

Ditt svar är för stort. Testa att förkorta en del av dina svar.

Journal *

If you already know where you will submit this paper (or if it is already submitted), please provide the journal name (if it is not JMIR, provide the journal name under "other")

- ( ) not submitted yet / unclear where I will submit this
- ( ) Journal of Medical Internet Research (JMIR)
- ( ) JMIR mHealth and UHealth
- ( ) JMIR Serious Games
- (•) JMIR Mental Health
- ( ) JMIR Public Health
- ( ) JMIR Formative Research
- ( ) Other JMIR sister journal
- ( ) Övrigt:

Is this a full powered effectiveness trial or a pilot/feasibility trial? *

- ( ) Pilot/feasibility
- (•) Fully powered

Manuscript tracking number *

If this is a JMIR submission, please provide the manuscript tracking number under "other" (The ms tracking number can be found in the submission acknowledgement email, or when you login as author in JMIR. If the paper is already published in JMIR, then the ms tracking number is the four-digit number at the end of the DOI, to be found at the bottom of each published article in JMIR)

- (•) no ms number (yet) / not (yet) submitted to / published in JMIR
- ( ) Övrigt:

TITLE AND ABSTRACT

1a) TITLE: Identification as a randomized trial in the title

Ditt svar är för stort. Testa att förkorta en del av dina svar.

1a) Does your paper address CONSORT item 1a? *

I.e does the title contain the phrase "Randomized Controlled Trial"? (if not, explain the reason under "other")

- (●) yes
- ( ) Övrigt:

1a-i) Identify the mode of delivery in the title

Identify the mode of delivery. Preferably use “web-based” and/or “mobile” and/or “electronic game” in the title. Avoid ambiguous terms like “online”, “virtual”, “interactive”. Use “Internet-based” only if Intervention includes non-web-based Internet components (e.g. email), use “computer-based” or “electronic” only if offline products are used. Use “virtual” only in the context of “virtual reality” (3-D worlds). Use “online” only in the context of “online support groups”. Complement or substitute product names with broader terms for the class of products (such as “mobile” or “smart phone” instead of “iphone”), especially if the application runs on different platforms.

	1	2	3	4	5	
subitem not at all important	○	○	○	○	○	essential

Does your paper address subitem 1a-i? *

Copy and paste relevant sections from manuscript title (include quotes in quotation marks "like this" to indicate direct quotes from your manuscript), or elaborate on this item by providing additional information not in the ms, or briefly explain why the item is not applicable/relevant for your study

"Internet-based"

1a-ii) Non-web-based components or important co-interventions in title

Mention non-web-based components or important co-interventions in title, if any (e.g., “with telephone support”).

	1	2	3	4	5	
subitem not at all important	○	○	○	○	○	essential

Does your paper address subitem 1a-ii?

Copy and paste relevant sections from manuscript title (include quotes in quotation marks "like this" to indicate direct quotes from your manuscript), or elaborate on this item by providing additional information not in the ms, or briefly explain why the item is not applicable/relevant for your study

No

Ditt svar är för stort. Testa att förkorta en del av dina svar.

1a-iii) Primary condition or target group in the title

Mention primary condition or target group in the title, if any (e.g., “for children with Type I Diabetes”) Example: A Web-based and Mobile Intervention with Telephone Support for Children with Type I Diabetes: Randomized Controlled Trial

	1	2	3	4	5	
subitem not at all important	○	○	○	○	○	essential

Does your paper address subitem 1a-iii? *

Copy and paste relevant sections from manuscript title (include quotes in quotation marks "like this" to indicate direct quotes from your manuscript), or elaborate on this item by providing additional information not in the ms, or briefly explain why the item is not applicable/relevant for your study

"for women with fear of birth"

1b) ABSTRACT: Structured summary of trial design, methods, results, and conclusions

NPT extension: Description of experimental treatment, comparator, care providers, centers, and blinding status.

1b-i) Key features/functionalities/components of the intervention and comparator in the METHODS section of the ABSTRACT

Mention key features/functionalities/components of the intervention and comparator in the abstract. If possible, also mention theories and principles used for designing the site. Keep in mind the needs of systematic reviewers and indexers by including important synonyms. (Note: Only report in the abstract what the main paper is reporting. If this information is missing from the main body of text, consider adding it)

	1	2	3	4	5	
subitem not at all important	○	○	○	○	○	essential

Ditt svar är för stort. Testa att förkorta en del av dina svar.

Does your paper address subitem 1b-i? *

Copy and paste relevant sections from the manuscript abstract (include quotes in quotation marks "like this" to indicate direct quotes from your manuscript), or elaborate on this item by providing additional information not in the ms, or briefly explain why the item is not applicable/relevant for your study

"The therapist guided ICBT intervention was inspired by the Unified protocol for transdiagnostic treatment of emotional disorders (UP), and consisted of eight treatment modules, and one module for postpartum follow up. The aim was to help participants observe and understand their FOB, and find new ways of coping with difficult thoughts and emotions. SC was offered in the three different study regions."

1b-ii) Level of human involvement in the METHODS section of the ABSTRACT

Clarify the level of human involvement in the abstract, e.g., use phrases like “fully automated” vs. “therapist/nurse/care provider/physician-assisted” (mention number and expertise of providers involved, if any). (Note: Only report in the abstract what the main paper is reporting. If this information is missing from the main body of text, consider adding it)

	1	2	3	4	5	
subitem not at all important	○	○	○	○	○	essential

Does your paper address subitem 1b-ii?

Copy and paste relevant sections from the manuscript abstract (include quotes in quotation marks "like this" to indicate direct quotes from your manuscript), or elaborate on this item by providing additional information not in the ms, or briefly explain why the item is not applicable/relevant for your study

"therapist guided"

Ditt svar är för stort. Testa att förkorta en del av dina svar.

1b-iii) Open vs. closed, web-based (self-assessment) vs. face-to-face assessments in the METHODS section of the ABSTRACT

Mention how participants were recruited (online vs. offline), e.g., from an open access website or from a clinic or a closed online user group (closed usergroup trial), and clarify if this was a purely web-based trial, or there were face-to-face components (as part of the intervention or for assessment). Clearly say if outcomes were self-assessed through questionnaires (as common in web-based trials). Note: In traditional offline trials, an open trial (open-label trial) is a type of clinical trial in which both the researchers and participants know which treatment is being administered. To avoid confusion, use “blinded” or “unblinded” to indicated the level of blinding instead of “open”, as “open” in web-based trials usually refers to “open access” (i.e. participants can self-enrol). (Note: Only report in the abstract what the main paper is reporting. If this information is missing from the main body of text, consider adding it)

	1	2	3	4	5	
subitem not at all important	○	○	○	○	○	essential

Does your paper address subitem 1b-iii?

Copy and paste relevant sections from the manuscript abstract (include quotes in quotation marks "like this" to indicate direct quotes from your manuscript), or elaborate on this item by providing additional information not in the ms, or briefly explain why the item is not applicable/relevant for your study

"Recruitment commenced at the ultrasound screening examination in gestational week 17-20." "The primary outcome was self-assessed levels of FOB, measured using the Fear of Birth Scale (FOBS). "

1b-iv) RESULTS section in abstract must contain use data

Report number of participants enrolled/assessed in each group, the use/uptake of the intervention (e.g., attrition/adherence metrics, use over time, number of logins etc.), in addition to primary/secondary outcomes. (Note: Only report in the abstract what the main paper is reporting. If this information is missing from the main body of text, consider adding it)

	1	2	3	4	5	
subitem not at all important	○	○	○	○	○	essential

Ditt svar är för stort. Testa att förkorta en del av dina svar.

Does your paper address subitem 1b-iv?

Copy and paste relevant sections from the manuscript abstract (include quotes in quotation marks "like this" to indicate direct quotes from your manuscript), or elaborate on this item by providing additional information not in the ms, or briefly explain why the item is not applicable/relevant for your study

"A total of 258 pregnant of women reporting clinically significant levels of FOB were included in the study (127 in the guided ICBT-group and 131 in the SC-group). Of the women randomized to guided ICBT, 103 (81%) commenced treatment, 60 (47%) moved on to the second module, and only 10% finished four modules or more. Levels of FOB did not differ between the intervention groups at post-intervention. At follow-up one year after birth, participants in the guided ICBT-group had significantly lower levels of FOB (U=3674.00, z=-1.97, P=.049, d=0.28, 95% CI -0.01-0.57). Using Linear Mixed Models Analysis a significant effect of time was found, with FOBS decreasing by 0.38 units per week, F(905) = 220.08, P=<.001, along with a significant interaction between time and intervention, showing a larger reduction in FOB in the guided ICBT-group over time, F(192.538) = 4.96, P=.03."

1b-v) CONCLUSIONS/DISCUSSION in abstract for negative trials

Conclusions/Discussions in abstract for negative trials: Discuss the primary outcome - if the trial is negative (primary outcome not changed), and the intervention was not used, discuss whether negative results are attributable to lack of uptake and discuss reasons. (Note: Only report in the abstract what the main paper is reporting. If this information is missing from the main body of text, consider adding it)

	1	2	3	4	5	
subitem not at all important	○	○	○	○	○	essential

Does your paper address subitem 1b-v?

Copy and paste relevant sections from the manuscript abstract (include quotes in quotation marks "like this" to indicate direct quotes from your manuscript), or elaborate on this item by providing additional information not in the ms, or briefly explain why the item is not applicable/relevant for your study

"FOB decreased over time in both intervention groups. Although the decrease was slightly larger in the guided ICBT-group, the effect of time alone was most evident."

INTRODUCTION

Ditt svar är för stort. Testa att förkorta en del av dina svar.

2a) In INTRODUCTION: Scientific background and explanation of rationale

2a-i) Problem and the type of system/solution

Describe the problem and the type of system/solution that is object of the study: intended as stand-alone intervention vs. incorporated in broader health care program? Intended for a particular patient population? Goals of the intervention, e.g., being more cost-effective to other interventions, replace or complement other solutions? (Note: Details about the intervention are provided in “Methods” under 5)

	1	2	3	4	5	
subitem not at all important	○	○	○	○	○	essential

Does your paper address subitem 2a-i? *

Copy and paste relevant sections from the manuscript (include quotes in quotation marks "like this" to indicate direct quotes from your manuscript), or elaborate on this item by providing additional information not in the ms, or briefly explain why the item is not applicable/relevant for your study

“To assess the efficacy of a guided Internet-based self-help program based on CBT (guided ICBT) compared with standard care (SC) on levels of FOB in late pregnancy and one year after birth in a sample of primiparous and multiparous women reporting clinically significant levels of FOB in Sweden.”

2a-ii) Scientific background, rationale: What is known about the (type of) system

Scientific background, rationale: What is known about the (type of) system that is the object of the study (be sure to discuss the use of similar systems for other conditions/diagnoses, if appropiate), motivation for the study, i.e. what are the reasons for and what is the context for this specific study, from which stakeholder viewpoint is the study performed, potential impact of findings [2]. Briefly justify the choice of the comparator.

	1	2	3	4	5	
subitem not at all important	○	○	○	○	○	essential

Ditt svar är för stort. Testa att förkorta en del av dina svar.

Does your paper address subitem 2a-ii? *

Copy and paste relevant sections from the manuscript (include quotes in quotation marks "like this" to indicate direct quotes from your manuscript), or elaborate on this item by providing additional information not in the ms, or briefly explain why the item is not applicable/relevant for your study

“There is no consensus on how FOB should be handled in clinical care. A few different treatment protocols have been evaluated in randomized controlled trials (RCT).” ... “Although generally showing some positive effects /.../ none of these studies have been convincing in reducing FOB. Given the apparent associations between FOB and measures of anxiety and depression, treatment protocols known to be efficacious in reducing fear, anxiety and depressive symptoms are thus important to explore.” ... “To date, cognitive behavior therapy (CBT) is the treatment of choice for most anxiety disorders [25-29]” “... transdiagnostic CBT treatment protocols have shown to be as efficacious as diagnosis-specific interventions for anxiety disorders”... “evaluations of interventions building on the principles and techniques of CBT but provided over the Internet also suggest equivalency with face-to-face CBT in terms of efficacy [34]”... “Although Internet-based self-help based on the principles of CBT could hold promise as a treatment alternative for women experiencing FOB, only one earlier study investigating the feasibility of such an approach has been published.” This was a non-randomized feasibility study reporting a within-group decrease in FOB from pre-intervention to post-intervention (Cohen’s d = 0.95).

2b) In INTRODUCTION: Specific objectives or hypotheses

Does your paper address CONSORT subitem 2b? *

Copy and paste relevant sections from the manuscript (include quotes in quotation marks "like this" to indicate direct quotes from your manuscript), or elaborate on this item by providing additional information not in the ms, or briefly explain why the item is not applicable/relevant for your study

“Thus, the primary aim of this RCT was to evaluate the efficacy of a guided Internet-based self-help program based on CBT (guided ICBT) compared with standard care (SC) on levels of FOB in late pregnancy and one year after birth in a sample of primiparous and multiparous women reporting clinically significant levels of FOB in Sweden.”

METHODS

Ditt svar är för stort. Testa att förkorta en del av dina svar.

3a) Description of trial design (such as parallel, factorial) including allocation ratio

Does your paper address CONSORT subitem 3a? *

Copy and paste relevant sections from the manuscript (include quotes in quotation marks "like this" to indicate direct quotes from your manuscript), or elaborate on this item by providing additional information not in the ms, or briefly explain why the item is not applicable/relevant for your study

“This multicenter randomized controlled trial with a parallel design was part of the U-CARE: pregnancy trial, comparing guided ICBT with SC for pregnant women reporting FOB [38].”

3b) Important changes to methods after trial commencement (such as eligibility criteria), with reasons

Does your paper address CONSORT subitem 3b? *

Copy and paste relevant sections from the manuscript (include quotes in quotation marks "like this" to indicate direct quotes from your manuscript), or elaborate on this item by providing additional information not in the ms, or briefly explain why the item is not applicable/relevant for your study

”About halftime through the project, the psychologists started to call each participant randomized to guided ICBT in order to optimize adherence and motivation.” Additional data collection via offline questionnaires was added at follow-up. “Data was collected at four time points” /.../ “via the U-CARE portal and offline questionnaires at follow up, one year after birth.”

3b-i) Bug fixes, Downtimes, Content Changes

Bug fixes, Downtimes, Content Changes: ehealth systems are often dynamic systems. A description of changes to methods therefore also includes important changes made on the intervention or comparator during the trial (e.g., major bug fixes or changes in the functionality or content) (5-iii) and other “unexpected events” that may have influenced study design such as staff changes, system failures/downtimes, etc. [2].

	1	2	3	4	5	
subitem not at all important	○	○	○	○	○	essential

Ditt svar är för stort. Testa att förkorta en del av dina svar.

Does your paper address subitem 3b-i?

Copy and paste relevant sections from the manuscript (include quotes in quotation marks "like this" to indicate direct quotes from your manuscript), or elaborate on this item by providing additional information not in the ms, or briefly explain why the item is not applicable/relevant for your study

None

4a) Eligibility criteria for participants

Does your paper address CONSORT subitem 4a? *

Copy and paste relevant sections from the manuscript (include quotes in quotation marks "like this" to indicate direct quotes from your manuscript), or elaborate on this item by providing additional information not in the ms, or briefly explain why the item is not applicable/relevant for your study

“Inclusion criteria were ongoing pregnancy in gestational week 17-20, a normal ultrasound screening examination, FOBS ≥ 60, mastery of the Swedish language and personal access to a mobile phone and computer with Internet connection.”

4a-i) Computer / Internet literacy

Computer / Internet literacy is often an implicit “de facto” eligibility criterion - this should be explicitly clarified.

	1	2	3	4	5	
subitem not at all important	○	○	○	○	○	essential

Does your paper address subitem 4a-i?

Copy and paste relevant sections from the manuscript (include quotes in quotation marks "like this" to indicate direct quotes from your manuscript), or elaborate on this item by providing additional information not in the ms, or briefly explain why the item is not applicable/relevant for your study

Participants were asked about their computer literacy at pre-intervention. Computer-literacy was high. In all, 10 women who had no access to a computer and/or were non-comfortable with using a computer were excluded.

Ditt svar är för stort. Testa att förkorta en del av dina svar.

4a-ii) Open vs. closed, web-based vs. face-to-face assessments:

Open vs. closed, web-based vs. face-to-face assessments: Mention how participants were recruited (online vs. offline), e.g., from an open access website or from a clinic, and clarify if this was a purely web-based trial, or there were face-to-face components (as part of the intervention or for assessment), i.e., to what degree got the study team to know the participant. In online-only trials, clarify if participants were quasi-anonymous and whether having multiple identities was possible or whether technical or logistical measures (e.g., cookies, email confirmation, phone calls) were used to detect/prevent these.

	1	2	3	4	5	
subitem not at all important	○	○	○	○	○	essential

Does your paper address subitem 4a-ii? *

Copy and paste relevant sections from the manuscript (include quotes in quotation marks "like this" to indicate direct quotes from your manuscript), or elaborate on this item by providing additional information not in the ms, or briefly explain why the item is not applicable/relevant for your study

“women attending the ultrasound screening examination in gestational week 17-20 were screened for possible identification of FOB.” ... “Before enrollment, eligible women were given information about the study by the research midwives.” “The guided ICBT program was delivered via a secure online platform, The U-CARE portal (www.u-care.se)”. “During the intervention, the participants were anonymous to their therapist.” Except a screening procedure taking place within routine antenatal care, this was initially a purely web-based trial. However, “About halftime through the project, the psychologists started to call each participant randomized to guided ICBT in order to optimize adherence and motivation. In total, 37 participants talked with their psychologist on the phone, while 15 did not respond despite several calls.”

4a-iii) Information giving during recruitment

Information given during recruitment. Specify how participants were briefed for recruitment and in the informed consent procedures (e.g., publish the informed consent documentation as appendix, see also item X26), as this information may have an effect on user self-selection, user expectation and may also bias results.

	1	2	3	4	5	
subitem not at all important	○	○	○	○	○	essential

Ditt svar är för stort. Testa att förkorta en del av dina svar.

Does your paper address subitem 4a-iii?

Copy and paste relevant sections from the manuscript (include quotes in quotation marks "like this" to indicate direct quotes from your manuscript), or elaborate on this item by providing additional information not in the ms, or briefly explain why the item is not applicable/relevant for your study

“Before enrollment, eligible women were given written and oral information about the study by the research midwives, and the women willing to participate gave their written informed consent.”

Before screening, women were given a leaflet with brief information about the study and contact details to the research team. A research midwife contacted women screening positively for fear of birth by phone. She informed the women about the study and they were able to ask questions. An information letter and a written consent form was sent to the women that were willing to participate. The information included the background and purpose of the study, a description of the methods for data collection, descriptions of the interventions (guided ICBT and SC) and the randomization procedure, how data was to be handled and the principle of confidentiality, the voluntariness of participation, and the responsibilities of the researchers along with contact information.

4b) Settings and locations where the data were collected

Does your paper address CONSORT subitem 4b? *

Copy and paste relevant sections from the manuscript (include quotes in quotation marks "like this" to indicate direct quotes from your manuscript), or elaborate on this item by providing additional information not in the ms, or briefly explain why the item is not applicable/relevant for your study

“The study was conducted at three study centers in Sweden, one university hospital with an annual rate of 4000 births, and two referral hospitals with an annual rate of 2800 and 1600 births respectively.”

4b-i) Report if outcomes were (self-)assessed through online questionnaires

Clearly report if outcomes were (self-)assessed through online questionnaires (as common in web-based trials) or otherwise.

	1	2	3	4	5	
subitem not at	○	○	○	○	○	essential

Ditt svar är för stort. Testa att förkorta en del av dina svar.

Does your paper address subitem 4b-i? *

Copy and paste relevant sections from the manuscript (include quotes in quotation marks "like this" to indicate direct quotes from your manuscript), or elaborate on this item by providing additional information not in the ms, or briefly explain why the item is not applicable/relevant for your study

“Data was collected through self-assessment questionnaires at four time points / …/ via the U-CARE portal”

4b-ii) Report how institutional affiliations are displayed

Report how institutional affiliations are displayed to potential participants [on ehealth media], as affiliations with prestigious hospitals or universities may affect volunteer rates, use, and reactions with regards to an intervention.(Not a required item – describe only if this may bias results)

	1	2	3	4	5	
subitem not at all important	○	○	○	○	○	essential

Does your paper address subitem 4b-ii?

Copy and paste relevant sections from the manuscript (include quotes in quotation marks "like this" to indicate direct quotes from your manuscript), or elaborate on this item by providing additional information not in the ms, or briefly explain why the item is not applicable/relevant for your study

No

5) The interventions for each group with sufficient details to allow replication, including how and when they were actually administered

5-i) Mention names, credential, affiliations of the developers, sponsors, and owners

Mention names, credential, affiliations of the developers, sponsors, and owners [6] (if authors/evaluators are owners or developer of the software, this needs to be declared in a “Conflict of interest” section or mentioned elsewhere in the manuscript).

	1	2	3	4	5	
subitem not at all important	○	○	○	○	○	essential

Ditt svar är för stort. Testa att förkorta en del av dina svar.

Does your paper address subitem 5-i?

Copy and paste relevant sections from the manuscript (include quotes in quotation marks "like this" to indicate direct quotes from your manuscript), or elaborate on this item by providing additional information not in the ms, or briefly explain why the item is not applicable/relevant for your study

“This multicenter randomized controlled trial with a parallel design was part of the U-CARE: pregnancy trial,”

The U-CARE: pregnancy trial is associated with the Uppsala University Psychosocial Care Programme (U-CARE), a government-funded project aimed at preventing and reducing emotional distress for patients with somatic diseases via the internet. Within the program, an internet platform called the U-CARE portal has been developed (www.u-care.se). The portal is used for randomization, data collection and interventions undertaken within the U-CARE program.

5-ii) Describe the history/development process

Describe the history/development process of the application and previous formative evaluations (e.g., focus groups, usability testing), as these will have an impact on adoption/use rates and help with interpreting results.

	1	2	3	4	5	
subitem not at all important	○	○	○	○	○	essential

Does your paper address subitem 5-ii?

Copy and paste relevant sections from the manuscript (include quotes in quotation marks "like this" to indicate direct quotes from your manuscript), or elaborate on this item by providing additional information not in the ms, or briefly explain why the item is not applicable/relevant for your study

When the study commenced, the U-CARE portal as such, was not yet evaluated. However, usability tests had been proceeding for several months before the study commenced.

“The guided ICBT intervention was inspired by the Unified protocol for transdiagnostic treatment of emotional disorders (UP)". The program was developed specifically for this study and had not been evaluated previously.

Ditt svar är för stort. Testa att förkorta en del av dina svar.

5-iii) Revisions and updating

Revisions and updating. Clearly mention the date and/or version number of the application/intervention (and comparator, if applicable) evaluated, or describe whether the intervention underwent major changes during the evaluation process, or whether the development and/or content was “frozen” during the trial. Describe dynamic components such as news feeds or changing content which may have an impact on the replicability of the intervention (for unexpected events see item 3b).

	1	2	3	4	5	
subitem not at all important	○	○	○	○	○	essential

Does your paper address subitem 5-iii?

Copy and paste relevant sections from the manuscript (include quotes in quotation marks "like this" to indicate direct quotes from your manuscript), or elaborate on this item by providing additional information not in the ms, or briefly explain why the item is not applicable/relevant for your study

The content of the intervention was “frozen” during the trial: "Once the study was launched, the methods used for data collection and online intervention were frozen and could not be changed."

5-iv) Quality assurance methods

Provide information on quality assurance methods to ensure accuracy and quality of information provided [1], if applicable.

	1	2	3	4	5	
subitem not at all important	○	○	○	○	○	essential

Does your paper address subitem 5-iv?

Copy and paste relevant sections from the manuscript (include quotes in quotation marks "like this" to indicate direct quotes from your manuscript), or elaborate on this item by providing additional information not in the ms, or briefly explain why the item is not applicable/relevant for your study

No

Ditt svar är för stort. Testa att förkorta en del av dina svar.

5-v) Ensure replicability by publishing the source code, and/or providing screenshots/screen-capture video, and/or providing flowcharts of the algorithms used

Ensure replicability by publishing the source code, and/or providing screenshots/screen-capture video, and/or providing flowcharts of the algorithms used. Replicability (i.e., other researchers should in principle be able to replicate the study) is a hallmark of scientific reporting.

	1	2	3	4	5	
subitem not at all important	○	○	○	○	○	essential

Does your paper address subitem 5-v?

Copy and paste relevant sections from the manuscript (include quotes in quotation marks "like this" to indicate direct quotes from your manuscript), or elaborate on this item by providing additional information not in the ms, or briefly explain why the item is not applicable/relevant for your study

“(www.u-care.se)”

In Textbox 1 we present an “Overview of the guided Internet-based self-help program based cognitive behavior therapy (guided ICBT).”

5-vi) Digital preservation

Digital preservation: Provide the URL of the application, but as the intervention is likely to change or disappear over the course of the years; also make sure the intervention is archived (Internet Archive, webcitation.org, and/or publishing the source code or screenshots/videos alongside the article). As pages behind login screens cannot be archived, consider creating demo pages which are accessible without login.

	1	2	3	4	5	
subitem not at all important	○	○	○	○	○	essential

Does your paper address subitem 5-vi?

Copy and paste relevant sections from the manuscript (include quotes in quotation marks "like this" to indicate direct quotes from your manuscript), or elaborate on this item by providing additional information not in the ms, or briefly explain why the item is not applicable/relevant for your study

"the U-CARE portal [39]"
Reference 39: "https://www.u-care.se/ Archived at http://www.webcitation.org/6xcDC4mLG"

Ditt svar är för stort. Testa att förkorta en del av dina svar.

5-vii) Access

Access: Describe how participants accessed the application, in what setting/context, if they had to pay (or were paid) or not, whether they had to be a member of specific group. If known, describe how participants obtained "access to the platform and Internet" [1]. To ensure access for editors/reviewers/readers, consider to provide a "backdoor" login account or demo mode for reviewers/readers to explore the application (also important for archiving purposes, see vi).

	1	2	3	4	5	
subitem not at all important	○	○	○	○	○	essential

Does your paper address subitem 5-vii? *

Copy and paste relevant sections from the manuscript (include quotes in quotation marks "like this" to indicate direct quotes from your manuscript), or elaborate on this item by providing additional information not in the ms, or briefly explain why the item is not applicable/relevant for your study

"The women who gave their consent received login details to a study specific website called the U-CARE portal (www.u-care.se)"
"The guided ICBT program was delivered via a secure online platform, The U-CARE portal, using double verification for log in."
The portal could be accessed from any computer. However, it could not be used from a telephone or tablet.

5-viii) Mode of delivery, features/functionalities/components of the intervention and comparator, and the theoretical framework

Describe mode of delivery, features/functionalities/components of the intervention and comparator, and the theoretical framework [6] used to design them (instructional strategy [1], behaviour change techniques, persuasive features, etc., see e.g., [7, 8] for terminology). This includes an in-depth description of the content (including where it is coming from and who developed it) [1]," whether [and how] it is tailored to individual circumstances and allows users to track their progress and receive feedback" [6]. This also includes a description of communication delivery channels and – if computer-mediated communication is a component – whether communication was synchronous or asynchronous [6]. It also includes information on presentation strategies [1], including page design principles, average amount of text on pages, presence of hyperlinks to other resources, etc. [1].

	1	2	3	4	5	
subitem not at all important	○	○	○	○	○	essential

Ditt svar är för stort. Testa att förkorta en del av dina svar.

Does your paper address subitem 5-viii? *

Copy and paste relevant sections from the manuscript (include quotes in quotation marks "like this" to indicate direct quotes from your manuscript), or elaborate on this item by providing additional information not in the ms, or briefly explain why the item is not applicable/relevant for your study

“The guided ICBT intervention was inspired by the Unified protocol for transdiagnostic treatment of emotional disorders (UP) /.../[42,43]."
“The aim of the guided ICBT intervention was to help the participants observe and understand their FOB, and to find new ways of coping with their anxiety. The self-help material was in Swedish and consisted of text material (81 downloadable pdf pages, including work sheets), audio files, photographs, and assignments relating to each part of the program. It was divided into eight treatment modules, and one module for postpartum follow up" ..."Although inspired by UP, each module was adapted to suit the current population."
“Textbox 1. Overview of the guided Internet-based self-help program based cognitive behavior therapy (guided ICBT)." In this textbos we describe the content of each module, incldmust assignments.

5-ix) Describe use parameters

Describe use parameters (e.g., intended “doses” and optimal timing for use). Clarify what instructions or recommendations were given to the user, e.g., regarding timing, frequency, heaviness of use, if any, or was the intervention used ad libitum.

	1	2	3	4	5	
subitem not at all important	○	○	○	○	○	essential

Does your paper address subitem 5-ix?

Copy and paste relevant sections from the manuscript (include quotes in quotation marks "like this" to indicate direct quotes from your manuscript), or elaborate on this item by providing additional information not in the ms, or briefly explain why the item is not applicable/relevant for your study

“Participants were recommended to complete one self-help module per week.”

Ditt svar är för stort. Testa att förkorta en del av dina svar.

5-x) Clarify the level of human involvement

Clarify the level of human involvement (care providers or health professionals, also technical assistance) in the e-intervention or as co-intervention (detail number and expertise of professionals involved, if any, as well as "type of assistance offered, the timing and frequency of the support, how it is initiated, and the medium by which the assistance is delivered". It may be necessary to distinguish between the level of human involvement required for the trial, and the level of human involvement required for a routine application outside of a RCT setting (discuss under item 21 – generalizability).

	1	2	3	4	5	
subitem not at all important	○	○	○	○	○	essential

Does your paper address subitem 5-x?

Copy and paste relevant sections from the manuscript (include quotes in quotation marks "like this" to indicate direct quotes from your manuscript), or elaborate on this item by providing additional information not in the ms, or briefly explain why the item is not applicable/relevant for your study

"When randomized to guided ICBT, participants were also randomized to one of two licensed psychologists, who guided them through the self-help program"
"The psychologists were active in the U-CARE portal three times a week, giving feedback to homework assignments, sending reminders, and answering text messages from the participants."

5-xi) Report any prompts/reminders used

Report any prompts/reminders used: Clarify if there were prompts (letters, emails, phone calls, SMS) to use the application, what triggered them, frequency etc. It may be necessary to distinguish between the level of prompts/reminders required for the trial, and the level of prompts/reminders for a routine application outside of a RCT setting (discuss under item 21 – generalizability).

	1	2	3	4	5	
subitem not at all important	○	○	○	○	○	essential

Does your paper address subitem 5-xi? *

Copy and paste relevant sections from the manuscript (include quotes in quotation marks "like this" to indicate direct quotes from your manuscript), or elaborate on this item by providing additional information not in the ms, or briefly explain why the item is not applicable/relevant for your study

"A welcome message was sent to each participant in the portal, along with a text message to their phone. Participants who did not log in or follow the treatment plan received reminders, both in the portal and via text messages, at 10 days and four weeks after randomization or their last login."

Ditt svar är för stort. Testa att förkorta en del av dina svar.

5-xii) Describe any co-interventions (incl. training/support)

Describe any co-interventions (incl. training/support): Clearly state any interventions that are provided in addition to the targeted eHealth intervention, as ehealth intervention may not be designed as stand-alone intervention. This includes training sessions and support [1]. It may be necessary to distinguish between the level of training required for the trial, and the level of training for a routine application outside of a RCT setting (discuss under item 21 – generalizability.

	1	2	3	4	5	
subitem not at all important	○	○	○	○	○	essential

Does your paper address subitem 5-xii? *

Copy and paste relevant sections from the manuscript (include quotes in quotation marks "like this" to indicate direct quotes from your manuscript), or elaborate on this item by providing additional information not in the ms, or briefly explain why the item is not applicable/relevant for your study

There were no co-interventions

6a) Completely defined pre-specified primary and secondary outcome measures, including how and when they were assessed

Does your paper address CONSORT subitem 6a? *

Copy and paste relevant sections from the manuscript (include quotes in quotation marks "like this" to indicate direct quotes from your manuscript), or elaborate on this item by providing additional information not in the ms, or briefly explain why the item is not applicable/relevant for your study

“The primary outcome measure for the present study was levels of FOB, measured in late pregnancy using the Fear of Birth Scale (FOBS) [11,41]."

6a-i) Online questionnaires: describe if they were validated for online use and apply CHERRIES items to describe how the questionnaires were designed/deployed

If outcomes were obtained through online questionnaires, describe if they were validated for online use and apply CHERRIES items to describe how the questionnaires were designed/deployed [9].

	1	2	3	4	5	
subitem not at all important	○	○	○	○	○	essential

Ditt svar är för stort. Testa att förkorta en del av dina svar.

Does your paper address subitem 6a-i?

Copy and paste relevant sections from manuscript text

The online questionnaires were not validated for online use.

6a-ii) Describe whether and how “use” (including intensity of use/dosage) was defined/measured/monitored

Describe whether and how “use” (including intensity of use/dosage) was defined/measured/monitored (logins, logfile analysis, etc.). Use/adoption metrics are important process outcomes that should be reported in any ehealth trial.

	1	2	3	4	5	
subitem not at all important	○	○	○	○	○	essential

Does your paper address subitem 6a-ii?

Copy and paste relevant sections from manuscript text

Use was measured by calculating number of opened treatment modules and login mean time in the portal. We did not use a cut point to differentiate between users and non-users.

"Table 2 shows the number of treatment modules opened by the participants in the guided ICBT-group. Of 127 participants allocated to this intervention, 103 commenced treatment. Among those, the mean time logged in the portal was 39.96 minutes (SD 49.88, range 1-244 minutes) or a mean of 13.21 minutes per opened module (SD 10.03, range 0.5-47). Feedback from participants or care providers regarding adherence to the SC could not be retrieved."

6a-iii) Describe whether, how, and when qualitative feedback from participants was obtained

Describe whether, how, and when qualitative feedback from participants was obtained (e.g., through emails, feedback forms, interviews, focus groups).

	1	2	3	4	5	
subitem not at all important	○	○	○	○	○	essential

Ditt svar är för stort. Testa att förkorta en del av dina svar.

Does your paper address subitem 6a-iii?

Copy and paste relevant sections from manuscript text

The participants were able to give feedback through text-messages in the online portal at any time during the intervention. After the end of the trial, we have interviewed participants in both the guided ICBT-group and standard care group. Two qualitative studies of their experiences of the trial and the interventions are now in preparation.

6b) Any changes to trial outcomes after the trial commenced, with reasons

Does your paper address CONSORT subitem 6b? *

Copy and paste relevant sections from the manuscript (include quotes in quotation marks "like this" to indicate direct quotes from your manuscript), or elaborate on this item by providing additional information not in the ms, or briefly explain why the item is not applicable/relevant for your study

No changes were made in the outcome measures.

7a) How sample size was determined

NPT: When applicable, details of whether and how the clustering by care provides or centers was addressed

7a-i) Describe whether and how expected attrition was taken into account when calculating the sample size

Describe whether and how expected attrition was taken into account when calculating the sample size.

	1	2	3	4	5	
subitem not at all important	○	○	○	○	○	essential

Ditt svar är för stort. Testa att förkorta en del av dina svar.

Does your paper address subitem 7a-i?

Copy and paste relevant sections from manuscript title (include quotes in quotation marks "like this" to indicate direct quotes from your manuscript), or elaborate on this item by providing additional information not in the ms, or briefly explain why the item is not applicable/relevant for your study

"The sample size was determined on a reduction in level of FOB, assessed in mid-pregnancy and one year after giving birth. The sample size of the present study was based on a Swedish study, where 59% of the women that had FOB during pregnancy reported no FOB one year postpartum [40]. With a 20% reduction of FOB, a two-sided test, a power of 0.80 and a level of significance of 5%, the power calculation showed that approximately 200 participants needed to be enrolled in the study [38]." In order to compensate for 20% attrition, we decided to include and randomize at least 250 women.

7b) When applicable, explanation of any interim analyses and stopping guidelines

Does your paper address CONSORT subitem 7b? *

Copy and paste relevant sections from the manuscript (include quotes in quotation marks "like this" to indicate direct quotes from your manuscript), or elaborate on this item by providing additional information not in the ms, or briefly explain why the item is not applicable/relevant for your study

Not applicable

8a) Method used to generate the random allocation sequence

NPT: When applicable, how care providers were allocated to each trial group

Does your paper address CONSORT subitem 8a? *

Copy and paste relevant sections from the manuscript (include quotes in quotation marks "like this" to indicate direct quotes from your manuscript), or elaborate on this item by providing additional information not in the ms, or briefly explain why the item is not applicable/relevant for your study

"participants were randomized by the U-CARE portal (1:1) to either guided ICBT or to SC". Randomization was done automatically by the U-CARE portal (1:1) to either guided ICBT or to SC without involvement by the researchers. Randomization was not restricted in any way. "When randomized to guided ICBT, participants were also randomized to one of two licensed psychologists, who guided them through the self-help program."

Ditt svar är för stort. Testa att förkorta en del av dina svar.

8b) Type of randomisation; details of any restriction (such as blocking and block size)

Does your paper address CONSORT subitem 8b? *

Copy and paste relevant sections from the manuscript (include quotes in quotation marks "like this" to indicate direct quotes from your manuscript), or elaborate on this item by providing additional information not in the ms, or briefly explain why the item is not applicable/relevant for your study

Randomization was done automatically by the U-CARE portal (1:1) to either guided ICBT or to SC without involvement by the researchers. Randomization was not restricted in any way.

9) Mechanism used to implement the random allocation sequence (such as sequentially numbered containers), describing any steps taken to conceal the sequence until interventions were assigned

Does your paper address CONSORT subitem 9? *

Copy and paste relevant sections from the manuscript (include quotes in quotation marks "like this" to indicate direct quotes from your manuscript), or elaborate on this item by providing additional information not in the ms, or briefly explain why the item is not applicable/relevant for your study

Participants were randomized automatically by the U-CARE portal directly after submitting the pre-intervention questionnaire.

10) Who generated the random allocation sequence, who enrolled participants, and who assigned participants to interventions

Does your paper address CONSORT subitem 10? *

Copy and paste relevant sections from the manuscript (include quotes in quotation marks "like this" to indicate direct quotes from your manuscript), or elaborate on this item by providing additional information not in the ms, or briefly explain why the item is not applicable/relevant for your study

Participants were initially recruited by health care staff at "the ultrasound screening examination". If meeting inclusion criteria, they were invited to t participate by two research midwives. After consenting to participate, participants were randomized automatically by the U-CARE portal without involvement of the researchers.

Ditt svar är för stort. Testa att förkorta en del av dina svar.

11a) If done, who was blinded after assignment to interventions (for example, participants, care providers, those assessing outcomes) and how

NPT: Whether or not administering co-interventions were blinded to group assignment

11a-i) Specify who was blinded, and who wasn’t

Specify who was blinded, and who wasn’t. Usually, in web-based trials it is not possible to blind the participants [1, 3] (this should be clearly acknowledged), but it may be possible to blind outcome assessors, those doing data analysis or those administering co-interventions (if any).

	1	2	3	4	5	
subitem not at all important	○	○	○	○	○	essential

Does your paper address subitem 11a-i? *

Copy and paste relevant sections from the manuscript (include quotes in quotation marks "like this" to indicate direct quotes from your manuscript), or elaborate on this item by providing additional information not in the ms, or briefly explain why the item is not applicable/relevant for your study

Neither participants nor care providers were blinded in this study.
"This nonblinded multicenter randomized controlled trial"
There was no outcome assessment other than self-report. Only the primal investigator had access to the participants self-reports during the trial.

11a-ii) Discuss e.g., whether participants knew which intervention was the “intervention of interest” and which one was the “comparator”

Informed consent procedures (4a-ii) can create biases and certain expectations - discuss e.g., whether participants knew which intervention was the “intervention of interest” and which one was the “comparator”.

	1	2	3	4	5	
subitem not at all important	○	○	○	○	○	essential

Ditt svar är för stort. Testa att förkorta en del av dina svar.

Does your paper address subitem 11a-ii?

Copy and paste relevant sections from the manuscript (include quotes in quotation marks "like this" to indicate direct quotes from your manuscript), or elaborate on this item by providing additional information not in the ms, or briefly explain why the item is not applicable/relevant for your study

The women received oral and written information about both standard care and the guided ICBT-intervention, as well as information about the randomization, before consenting to participate. In the information it is stated that it is unknown which treatment is better.

11b) If relevant, description of the similarity of interventions

(this item is usually not relevant for ehealth trials as it refers to similarity of a placebo or sham intervention to a active medication/intervention)

Does your paper address CONSORT subitem 11b? *

Copy and paste relevant sections from the manuscript (include quotes in quotation marks "like this" to indicate direct quotes from your manuscript), or elaborate on this item by providing additional information not in the ms, or briefly explain why the item is not applicable/relevant for your study

Not applicable.

12a) Statistical methods used to compare groups for primary and secondary outcomes

NPT: When applicable, details of whether and how the clustering by care providers or centers was addressed

Does your paper address CONSORT subitem 12a? *

Copy and paste relevant sections from the manuscript (include quotes in quotation marks "like this" to indicate direct quotes from your manuscript), or elaborate on this item by providing additional information not in the ms, or briefly explain why the item is not applicable/relevant for your study

“Linear Mixed Model analyses" and "Mann-Whitney U test" were used to compare intervention groups. WE also calculated "Between- and within-group effect sizes (Cohen’s d) and their 95% confidence intervals" on " both observed and estimated data."

Ditt svar är för stort. Testa att förkorta en del av dina svar.

12a-i) Imputation techniques to deal with attrition / missing values

Imputation techniques to deal with attrition / missing values: Not all participants will use the intervention/comparator as intended and attrition is typically high in ehealth trials. Specify how participants who did not use the application or dropped out from the trial were treated in the statistical analysis (a complete case analysis is strongly discouraged, and simple imputation techniques such as LOCF may also be problematic [4]).

	1	2	3	4	5	
subitem not at all important	○	○	○	○	○	essential

Does your paper address subitem 12a-i? *

Copy and paste relevant sections from the manuscript (include quotes in quotation marks "like this" to indicate direct quotes from your manuscript), or elaborate on this item by providing additional information not in the ms, or briefly explain why the item is not applicable/relevant for your study

“Building on a likelihood-based approach, Linear Mixed Models analysis makes use of all available data, and produces unbiased parameter estimates under the assumption of data being missing at random, making it suitable for intention-to-treat analyses in longitudinal studies with data missing at random [52-54].”

12b) Methods for additional analyses, such as subgroup analyses and adjusted analyses

Does your paper address CONSORT subitem 12b? *

Copy and paste relevant sections from the manuscript (include quotes in quotation marks "like this" to indicate direct quotes from your manuscript), or elaborate on this item by providing additional information not in the ms, or briefly explain why the item is not applicable/relevant for your study

“Clinically significant reduction of FOB was calculated and defined by a cut point two standard deviations below the pre-intervention mean of the group [56]. Differences in the rate of treatment responders between the intervention groups were compared using Pearson’s chi-square test.“

X26) REB/IRB Approval and Ethical Considerations [recommended as subheading under "Methods"] (not a CONSORT item)

Ditt svar är för stort. Testa att förkorta en del av dina svar.

X26-i) Comment on ethics committee approval

	1	2	3	4	5	
subitem not at all important	○	○	○	○	○	essential

Does your paper address subitem X26-i?

Copy and paste relevant sections from the manuscript (include quotes in quotation marks "like this" to indicate direct quotes from your manuscript), or elaborate on this item by providing additional information not in the ms, or briefly explain why the item is not applicable/relevant for your study

This study ...”was approved by the Regional Ethical Review Board in Uppsala (No. 2013/209)”

x26-ii) Outline informed consent procedures

Outline informed consent procedures e.g., if consent was obtained offline or online (how? Checkbox, etc.?), and what information was provided (see 4a-ii). See [6] for some items to be included in informed consent documents.

	1	2	3	4	5	
subitem not at all important	○	○	○	○	○	essential

Does your paper address subitem X26-ii?

Copy and paste relevant sections from the manuscript (include quotes in quotation marks "like this" to indicate direct quotes from your manuscript), or elaborate on this item by providing additional information not in the ms, or briefly explain why the item is not applicable/relevant for your study

Two research midwives contacted women screening positively for fear of birth by phone. They informed the women about the study and invited them to participate. Women willing to participate were sent written information and a consent form by surface mail. The information included information about SC and ICBT, the online portal, randomization, pre- and post-intervention and follow-up questionnaires, duration of intervention, that participation was voluntary, confidentiality and contact persons for the study. Only women returning a written consent form were included in the study.

Ditt svar är för stort. Testa att förkorta en del av dina svar.

X26-iii) Safety and security procedures

Safety and security procedures, incl. privacy considerations, and any steps taken to reduce the likelihood or detection of harm (e.g., education and training, availability of a hotline)

	1	2	3	4	5	
subitem not at all important	○	○	○	○	○	essential

Does your paper address subitem X26-iii?

Copy and paste relevant sections from the manuscript (include quotes in quotation marks "like this" to indicate direct quotes from your manuscript), or elaborate on this item by providing additional information not in the ms, or briefly explain why the item is not applicable/relevant for your study

Ditt svar

RESULTS

13a) For each group, the numbers of participants who were randomly assigned, received intended treatment, and were analysed for the primary outcome

NPT: The number of care providers or centers performing the intervention in each group and the number of patients treated by each care provider in each center

Ditt svar är för stort. Testa att förkorta en del av dina svar.

Does your paper address CONSORT subitem 13a? *

Copy and paste relevant sections from the manuscript (include quotes in quotation marks "like this" to indicate direct quotes from your manuscript), or elaborate on this item by providing additional information not in the ms, or briefly explain why the item is not applicable/relevant for your study

”127 were allocated to guided ICBT and 131 to SC. The full CONSORT flowchart is presented in Figure 1.” This was a pure intention-to-treat study. All women randomized to either intervention were included in the analysis of the primary outcome – using linear mixed effect model analysis. We also present both observed and estimated for both groups, along with within- and between groups effect sizes based on both observed and estimated data. We did not define any cut off to define who “received” the guided ICBT intervention and “Feedback from participants or care providers regarding adherence to the SC could not be retrieved.”
Standard care: “The study was conducted at three study centers in Sweden”
Guided ICBT: “When randomized to guided ICBT, participants were also randomized to one of two licensed psychologists, who guided them through the self-help program.” The number of patients treated by each psychologist is not presented in the manuscript.

13b) For each group, losses and exclusions after randomisation, together with reasons

Does your paper address CONSORT subitem 13b? (NOTE: Preferably, this is shown in a CONSORT flow diagram) *

Copy and paste relevant sections from the manuscript (include quotes in quotation marks "like this" to indicate direct quotes from your manuscript), or elaborate on this item by providing additional information not in the ms, or briefly explain why the item is not applicable/relevant for your study

“The full CONSORT flowchart is presented in Figure 1.”

13b-i) Attrition diagram

Strongly recommended: An attrition diagram (e.g., proportion of participants still logging in or using the intervention/comparator in each group plotted over time, similar to a survival curve) or other figures or tables demonstrating usage/dose/engagement.

	1	2	3	4	5	
subitem not at all important	○	○	○	○	○	essential

Ditt svar är för stort. Testa att förkorta en del av dina svar.

Does your paper address subitem 13b-i?

Copy and paste relevant sections from the manuscript or cite the figure number if applicable (include quotes in quotation marks "like this" to indicate direct quotes from your manuscript), or elaborate on this item by providing additional information not in the ms, or briefly explain why the item is not applicable/relevant for your study

“Table 2 shows the number of treatment modules opened by the participants in the guided ICBT-group. Of 127 participants allocated to this intervention, 103 commenced treatment. Among those, the mean time logged in the portal was 39.96 minutes (SD 49.88, range 1-244 minutes) or 13.21 minutes per opened module (SD 10.03, range 0.5-47). Feedback from participants or care providers regarding adherence to the SC could not be retrieved.”

14a) Dates defining the periods of recruitment and follow-up

Does your paper address CONSORT subitem 14a? *

Copy and paste relevant sections from the manuscript (include quotes in quotation marks "like this" to indicate direct quotes from your manuscript), or elaborate on this item by providing additional information not in the ms, or briefly explain why the item is not applicable/relevant for your study

“Between February 2014 and February 2015, women attending the ultrasound screening examination in gestational week 17-20 were screened for possible identification of FOB.”

“Data was collected at four time points: (1) at the ultrasound screening examination in gestational week 17-20, (2) via the U-CARE portal at pre-intervention in gestational week 20–25, (3) via the U-CARE portal at post-intervention in gestational week 30 and 36, (4) via the U-CARE portal and by at follow up, one year after birth.”

14a-i) Indicate if critical “secular events” fell into the study period

Indicate if critical “secular events” fell into the study period, e.g., significant changes in Internet resources available or “changes in computer hardware or Internet delivery resources”

	1	2	3	4	5	
subitem not at all important	○	○	○	○	○	essential

Ditt svar är för stort. Testa att förkorta en del av dina svar.

Does your paper address subitem 14a-i?

Copy and paste relevant sections from the manuscript (include quotes in quotation marks "like this" to indicate direct quotes from your manuscript), or elaborate on this item by providing additional information not in the ms, or briefly explain why the item is not applicable/relevant for your study

None

14b) Why the trial ended or was stopped (early)

Does your paper address CONSORT subitem 14b? *

Copy and paste relevant sections from the manuscript (include quotes in quotation marks "like this" to indicate direct quotes from your manuscript), or elaborate on this item by providing additional information not in the ms, or briefly explain why the item is not applicable/relevant for your study

The trial was not ended or stopped early.

15) A table showing baseline demographic and clinical characteristics for each group

NPT: When applicable, a description of care providers (case volume, qualification, expertise, etc.) and centers (volume) in each group

Does your paper address CONSORT subitem 15? *

Copy and paste relevant sections from the manuscript (include quotes in quotation marks "like this" to indicate direct quotes from your manuscript), or elaborate on this item by providing additional information not in the ms, or briefly explain why the item is not applicable/relevant for your study

“Table 1 presents the pre-intervention characteristics of the participants in the study sample.”
Guided ICBT: “two licensed clinical psychologists" working specifically in the
Standard care: “Depending on what study center the participant belonged to, the SC started either in the next meeting with the antenatal midwife, or after referral to a counselling midwife or a psycho-social unit [38].”

15-i) Report demographics associated with digital divide issues

In ehealth trials it is particularly important to report demographics associated with digital divide issues, such as age, education, gender, social-economic status, computer/Internet/ehealth literacy of the participants, if known.

1 2 3 4 5

subitem not at [illegible]

Ditt svar är för stort. Testa att förkorta en del av dina svar.

Does your paper address subitem 15-i? *

Copy and paste relevant sections from the manuscript (include quotes in quotation marks "like this" to indicate direct quotes from your manuscript), or elaborate on this item by providing additional information not in the ms, or briefly explain why the item is not applicable/relevant for your study

All of the demographics of special interest, including computer literacy, are provided in Table 1, “Characteristics of the participants at pre-intervention”.

16) For each group, number of participants (denominator) included in each analysis and whether the analysis was by original assigned groups

16-i) Report multiple “denominators” and provide definitions

Report multiple “denominators” and provide definitions: Report N’s (and effect sizes) “across a range of study participation [and use] thresholds” [1], e.g., N exposed, N consented, N used more than x times, N used more than y weeks, N participants “used” the intervention/comparator at specific pre-defined time points of interest (in absolute and relative numbers per group). Always clearly define “use” of the intervention.

	1	2	3	4	5	
subitem not at all important	○	○	○	○	○	essential

Does your paper address subitem 16-i? *

Copy and paste relevant sections from the manuscript (include quotes in quotation marks "like this" to indicate direct quotes from your manuscript), or elaborate on this item by providing additional information not in the ms, or briefly explain why the item is not applicable/relevant for your study

The CONSORT flow-chart (Figure 1) describes the number of women screened, contacted, randomized etc., along with reasons for exclusion. “Table 2 shows the number of treatment modules opened by the participants in the guided ICBT-group." “Observed and estimated means and standard deviations of the primary outcome measure are plotted in Figure 2, and presented in Tables 3 and 4, along with the within-group effect sizes (Cohen’s d), and 95% confidence intervals.” Besides the mean and standard deviations for each intervention group at each timepoint, the table of observed means (Table 3) also include the number of respondents at each timepoint, and the pre-intervention mean and standard deviation on FOBS of the participants responding at post-intervention and follow-up.

Ditt svar är för stort. Testa att förkorta en del av dina svar.

16-ii) Primary analysis should be intent-to-treat

Primary analysis should be intent-to-treat, secondary analyses could include comparing only “users”, with the appropriate caveats that this is no longer a randomized sample (see 18-i).

	1	2	3	4	5	
subitem not at all important	○	○	○	○	○	essential

Does your paper address subitem 16-ii?

Copy and paste relevant sections from the manuscript (include quotes in quotation marks "like this" to indicate direct quotes from your manuscript), or elaborate on this item by providing additional information not in the ms, or briefly explain why the item is not applicable/relevant for your study

“In this intention-to-treat study"

17a) For each primary and secondary outcome, results for each group, and the estimated effect size and its precision (such as 95% confidence interval)

Does your paper address CONSORT subitem 17a? *

Copy and paste relevant sections from the manuscript (include quotes in quotation marks "like this" to indicate direct quotes from your manuscript), or elaborate on this item by providing additional information not in the ms, or briefly explain why the item is not applicable/relevant for your study

“Observed and estimated means and standard deviations of the primary outcome measure are plotted in Figure 2, and presented in Tables 3 and 4, along with the within-group effect sizes (Cohen’s d), and 95% confidence intervals.”

17a-i) Presentation of process outcomes such as metrics of use and intensity of use

In addition to primary/secondary (clinical) outcomes, the presentation of process outcomes such as metrics of use and intensity of use (dose, exposure) and their operational definitions is critical. This does not only refer to metrics of attrition (13-b) (often a binary variable), but also to more continuous exposure metrics such as “average session length”. These must be accompanied by a technical description how a metric like a “session” is defined (e.g., timeout after idle time) [1] (report under item 6a).

	1	2	3	4	5	
subitem not at all important	○	○	○	○	○	essential

Ditt svar är för stort. Testa att förkorta en del av dina svar.

Does your paper address subitem 17a-i?

Copy and paste relevant sections from the manuscript (include quotes in quotation marks "like this" to indicate direct quotes from your manuscript), or elaborate on this item by providing additional information not in the ms, or briefly explain why the item is not applicable/relevant for your study

Guided iCBT: “Of 127 participants allocated to this intervention, 103 commenced treatment. Among those, the mean time logged in the portal was 39.96 minutes (SD 49.88, range 1-244 minutes) or 13.21 minutes per opened module (SD 10.03, range 0.5-47).”

17b) For binary outcomes, presentation of both absolute and relative effect sizes is recommended

Does your paper address CONSORT subitem 17b? *

Copy and paste relevant sections from the manuscript (include quotes in quotation marks "like this" to indicate direct quotes from your manuscript), or elaborate on this item by providing additional information not in the ms, or briefly explain why the item is not applicable/relevant for your study

Not applicable.

18) Results of any other analyses performed, including subgroup analyses and adjusted analyses, distinguishing pre-specified from exploratory

Does your paper address CONSORT subitem 18? *

Copy and paste relevant sections from the manuscript (include quotes in quotation marks "like this" to indicate direct quotes from your manuscript), or elaborate on this item by providing additional information not in the ms, or briefly explain why the item is not applicable/relevant for your study

“In line with recommendations by Jacobsen and Truax [56], the cut point for responding to treatment was set to FOBS ≤ 38. At post-intervention a significantly higher proportion of the participants in the SC-group scored below this cut point, 29 (22.1%) as compared to 11 (8.7%) in the guided ICBT-group, $\chi 2$ (1) = 8.94, p = .003. At follow-up, the groups did not differ significantly, with 44 (34.6%) of the participants who were allocated to guided ICBT and 37 (28.2%) of participants in the SC-group reaching below this cut point.”
No sub-group analyses have been performed.

Ditt svar är för stort. Testa att förkorta en del av dina svar.

18-i) Subgroup analysis of comparing only users

A subgroup analysis of comparing only users is not uncommon in ehealth trials, but if done, it must be stressed that this is a self-selected sample and no longer an unbiased sample from a randomized trial (see 16-iii).

	1	2	3	4	5	
subitem not at all important	○	○	○	○	○	essential

Does your paper address subitem 18-i?

Copy and paste relevant sections from the manuscript (include quotes in quotation marks "like this" to indicate direct quotes from your manuscript), or elaborate on this item by providing additional information not in the ms, or briefly explain why the item is not applicable/relevant for your study

Given the overall low adherence to treatment no sub-group analyses comparing users only could be performed.

19) All important harms or unintended effects in each group

(for specific guidance see CONSORT for harms)

Does your paper address CONSORT subitem 19? *

Copy and paste relevant sections from the manuscript (include quotes in quotation marks "like this" to indicate direct quotes from your manuscript), or elaborate on this item by providing additional information not in the ms, or briefly explain why the item is not applicable/relevant for your study

“Although all participants scored above the clinical cut point for FOB at screening (FOBS ≥ 60), 52 of the participants (20.2%) scored below this cut point at pre-intervention.” ... “Unexpectedly, in 20% of the participants, we saw a reduction of FOB below the inclusion criteria cut point already before randomization and the introduction of any planned intervention. Although possibly an effect of the passage of time alone, this reduction might also be related to participants talking to a research midwife on the phone in order to be included in the study, or simply be due to statistical regression to the mean”.

Ditt svar är för stort. Testa att förkorta en del av dina svar.

19-i) Include privacy breaches, technical problems

Include privacy breaches, technical problems. This does not only include physical “harm” to participants, but also incidents such as perceived or real privacy breaches [1], technical problems, and other unexpected/unintended incidents. “Unintended effects” also includes unintended positive effects [2].

	1	2	3	4	5	
subitem not at all important	○	○	○	○	○	essential

Does your paper address subitem 19-i?

Copy and paste relevant sections from the manuscript (include quotes in quotation marks "like this" to indicate direct quotes from your manuscript), or elaborate on this item by providing additional information not in the ms, or briefly explain why the item is not applicable/relevant for your study

Technical problems occurred from time to time, however none threatening the privacy of the participants. Technical problems such as problems logging in, problems when reporting assignments, problems in opening files etc. were referred to the technical support group (available by phone or email). When this study was conducted, the U-CARE portal did not support the use of phones or tablets, which to some participants was reported as problematic.

19-ii) Include qualitative feedback from participants or observations from staff/researchers

Include qualitative feedback from participants or observations from staff/researchers, if available, on strengths and shortcomings of the application, especially if they point to unintended/unexpected effects or uses. This includes (if available) reasons for why people did or did not use the application as intended by the developers.

	1	2	3	4	5	
subitem not at all important	○	○	○	○	○	essential

Does your paper address subitem 19-ii?

Copy and paste relevant sections from the manuscript (include quotes in quotation marks "like this" to indicate direct quotes from your manuscript), or elaborate on this item by providing additional information not in the ms, or briefly explain why the item is not applicable/relevant for your study

Two qualitative studies focusing specifically on the experiences of women randomized to each treatment arm are in preparation.

DISCUSSION

Ditt svar är för stort. Testa att förkorta en del av dina svar.

22) Interpretation consistent with results, balancing benefits and harms, and considering other relevant evidence

NPT: In addition, take into account the choice of the comparator, lack of or partial blinding, and unequal expertise of care providers or centers in each group

22-i) Restate study questions and summarize the answers suggested by the data, starting with primary outcomes and process outcomes (use)

Restate study questions and summarize the answers suggested by the data, starting with primary outcomes and process outcomes (use).

	1	2	3	4	5	
subitem not at all important	○	○	○	○	○	essential

Does your paper address subitem 22-i? *

Copy and paste relevant sections from the manuscript (include quotes in quotation marks "like this" to indicate direct quotes from your manuscript), or elaborate on this item by providing additional information not in the ms, or briefly explain why the item is not applicable/relevant for your study

“In the present study, the level of FOB was found to decrease over time in both groups, generally with medium within-group effect sizes during pregnancy and large effect sizes from mid-pregnancy to one year postpartum.” ... “In the linear mixed model analyses, a small, yet significant, interaction between time and treatment was found, indicating that over time FOB decreased slightly more among participants allocated to guided ICBT. This effect was most evident at follow-up one year after birth, when participants in the guided ICBT group had significantly lower levels of FOB, however still with a small between-group effect size (d = 0.28).”

22-ii) Highlight unanswered new questions, suggest future research

Highlight unanswered new questions, suggest future research.

	1	2	3	4	5	
subitem not at all important	○	○	○	○	○	essential

Ditt svar är för stort. Testa att förkorta en del av dina svar.

Does your paper address subitem 22-ii?

Copy and paste relevant sections from the manuscript (include quotes in quotation marks "like this" to indicate direct quotes from your manuscript), or elaborate on this item by providing additional information not in the ms, or briefly explain why the item is not applicable/relevant for your study

“The challenge in future research will be to find an intervention that is both well-accepted by pregnant women and effective in reducing FOB. Considering the strong evidence for CBT in treating anxiety, cognitive and behavioral interventions should not be ruled out at this early point. However, in order to enhance credibility among pregnant women and caregivers, we need to learn more about the experiences of women participating in different intervention programs, and make adjustments in the treatment format and structure based on their views. Instead of comparing different treatment interventions it might be more fruitful to integrate existing and well-accepted midwife counselling with CBT interventions, acknowledging the need of individual tailoring.”

20) Trial limitations, addressing sources of potential bias, imprecision, and, if relevant, multiplicity of analyses

20-i) Typical limitations in ehealth trials

Typical limitations in ehealth trials: Participants in ehealth trials are rarely blinded. Ehealth trials often look at a multiplicity of outcomes, increasing risk for a Type I error. Discuss biases due to non-use of the intervention/usability issues, biases through informed consent procedures, unexpected events.

	1	2	3	4	5	
subitem not at all important	○	○	○	○	○	essential

Ditt svar är för stort. Testa att förkorta en del av dina svar.

Does your paper address subitem 20-i? *

Copy and paste relevant sections from the manuscript (include quotes in quotation marks "like this" to indicate direct quotes from your manuscript), or elaborate on this item by providing additional information not in the ms, or briefly explain why the item is not applicable/relevant for your study

This study was non-blinded. With only one outcome measure, the risk for Type I errors from multiplicity of outcomes is not applicable. Non-use of the guided ICBT was extensive and has been discussed within the limitations section, e.g. "In the guided ICBT-group, a very low treatment adherence is obvious. Unfortunately, it is difficult to know all the reasons participants had for not engaging in their ICBT. Some participants reported... [examples are given]". /.../ "it seems likely to assume that the guided ICBT was not a well-accepted intervention in this sample." /.../ "There are several possible reasons for this poor adherence, potentially relating to expectations and care preferences in the population, the process of inclusion and exclusion of participants, lack of pre-intervention assessment of individual needs, issues relating to the online portal, instructions and reminders not being sufficient, the treatment format, or the self-help material not meeting the expectations of the participants."

21) Generalisability (external validity, applicability) of the trial findings

NPT: External validity of the trial findings according to the intervention, comparators, patients, and care providers or centers involved in the trial

21-i) Generalizability to other populations

Generalizability to other populations: In particular, discuss generalizability to a general Internet population, outside of a RCT setting, and general patient population, including applicability of the study results for other organizations

	1	2	3	4	5	
subitem not at all important	○	○	○	○	○	essential

Ditt svar är för stort. Testa att förkorta en del av dina svar.

Does your paper address subitem 21-i?

Copy and paste relevant sections from the manuscript (include quotes in quotation marks "like this" to indicate direct quotes from your manuscript), or elaborate on this item by providing additional information not in the ms, or briefly explain why the item is not applicable/relevant for your study

“...the naturalistic setting of the study, and the representativeness of the sample are important for the generalization of results. When comparing the participants in our study with the general birthing population, few differences were found. The mean ages, the percentages of women living with a partner and education levels are all very similar to the official statistics of Sweden [68]. Since women with insufficient knowledge in the Swedish language were excluded in the present study, the results are not possible to generalize to this population.”

21-ii) Discuss if there were elements in the RCT that would be different in a routine application setting

Discuss if there were elements in the RCT that would be different in a routine application setting (e.g., prompts/reminders, more human involvement, training sessions or other co-interventions) and what impact the omission of these elements could have on use, adoption, or outcomes if the intervention is applied outside of a RCT setting.

	1	2	3	4	5	
subitem not at all important	○	○	○	○	○	essential

Does your paper address subitem 21-ii?

Copy and paste relevant sections from the manuscript (include quotes in quotation marks "like this" to indicate direct quotes from your manuscript), or elaborate on this item by providing additional information not in the ms, or briefly explain why the item is not applicable/relevant for your study

Given the low usage of the guided ICBT- treatment, the intervention is not recommended to be applied in routine care at this point.

OTHER INFORMATION

23) Registration number and name of trial registry

Ditt svar är för stort. Testa att förkorta en del av dina svar.

Does your paper address CONSORT subitem 23? *

Copy and paste relevant sections from the manuscript (include quotes in quotation marks "like this" to indicate direct quotes from your manuscript), or elaborate on this item by providing additional information not in the ms, or briefly explain why the item is not applicable/relevant for your study

ClinicalTrials.gov (NCT02306434)

24) Where the full trial protocol can be accessed, if available

Does your paper address CONSORT subitem 24? *

Cite a Multimedia Appendix, other reference, or copy and paste relevant sections from the manuscript (include quotes in quotation marks "like this" to indicate direct quotes from your manuscript), or elaborate on this item by providing additional information not in the ms, or briefly explain why the item is not applicable/relevant for your study

Ternström E, Hildingsson I, Haines H, Karlström A, Sundin Ö, Ekdahl J, Segeblad B, Larsson B, Rondung E, Rubertsson C. A randomized controlled study comparing internet-based cognitive behavioral therapy and counselling by standard care for fear of birth – A study protocol. Sex Reprod Healthc 2017 Oct 1;13(Supplement C):75–82. PMID:28844361

25) Sources of funding and other support (such as supply of drugs), role of funders

Does your paper address CONSORT subitem 25? *

Copy and paste relevant sections from the manuscript (include quotes in quotation marks "like this" to indicate direct quotes from your manuscript), or elaborate on this item by providing additional information not in the ms, or briefly explain why the item is not applicable/relevant for your study

This study was funded by Institutional Grants from Uppsala University.

X27) Conflicts of Interest (not a CONSORT item)

Ditt svar är för stort. Testa att förkorta en del av dina svar.

X27-i) State the relation of the study team towards the system being evaluated

In addition to the usual declaration of interests (financial or otherwise), also state the relation of the study team towards the system being evaluated, i.e., state if the authors/evaluators are distinct from or identical with the developers/sponsors of the intervention.

	1	2	3	4	5	
subitem not at all important	○	○	○	○	○	essential

Does your paper address subitem X27-i?

Copy and paste relevant sections from the manuscript (include quotes in quotation marks "like this" to indicate direct quotes from your manuscript), or elaborate on this item by providing additional information not in the ms, or briefly explain why the item is not applicable/relevant for your study

The authors/evaluators of this study have participated in the design of the guided ICBT intervention.

About the CONSORT EHEALTH checklist

As a result of using this checklist, did you make changes in your manuscript? *

- ○ yes, major changes
- ◉ yes, minor changes
- ○ no

What were the most important changes you made as a result of using this checklist?

Despite no statistical difference, we included separate demographic information for each intervention-group. We also updated the abstract.

How much time did you spend on going through the checklist INCLUDING making changes in your manuscript *

5 work days

Ditt svar är för stort. Testa att förkorta en del av dina svar.

As a result of using this checklist, do you think your manuscript has improved? *

- (x) yes
- ( ) no
- ( ) Övrigt:

Would you like to become involved in the CONSORT EHEALTH group?

This would involve for example becoming involved in participating in a workshop and writing an "Explanation and Elaboration" document

- ( ) yes
- (x) no
- ( ) Övrigt:

Any other comments or questions on CONSORT EHEALTH

Ditt svar

STOP - Save this form as PDF before you click submit

To generate a record that you filled in this form, we recommend to generate a PDF of this page (on a Mac, simply select "print" and then select "print as PDF") before you submit it.

When you submit your (revised) paper to JMIR, please upload the PDF as supplementary file.

Don't worry if some text in the textboxes is cut off, as we still have the complete information in our database. Thank you!

Final step: Click submit !

Click submit so we have your answers in our database!

SKICKA

Ditt svar är för stort. Testa att förkorta en del av dina svar.

Det här innehållet har varken skapats eller godkänts av Google. Anmäl otillåten användning - Användarvillkor - Ytterligare villkor

Google Formulär

Ditt svar är för stort. Testa att förkorta en del av dina svar.
